# Adoptive NK cell transfer confers neuroprotection by attenuating neuroinflammation and alpha-synuclein pathology in a mouse model of synucleinopathy

**DOI:** 10.1186/s44477-025-00006-9

**Published:** 2025-11-27

**Authors:** Adetutu Adebowale, Jung Ha Byun, Jaegwon Chung, Hyun Joon Lee, Declan Gresham, Jina Lim, Jae-Kyung Lee

**Affiliations:** 1 Department of Physiology and Pharmacology, College of Veterinary Medicine, University of Georgia, 501 D.W. Brooks Drive, Athens, GA 30602 USA; 2 Intergrative Physiology and Pharmacology Program, University of Georgia, Athens, GA USA; 3 Neuroscience Program, University of Georgia, Athens, GA USA

**Keywords:** Parkinson’s disease, NK cell, Neuroinflammation, Alpha-synuclein, Dopaminergic, Lewy pathology

## Abstract

**Background:**

Natural killer (NK) cells are key effector lymphoid cells involved in both innate and adaptive immunity and are capable of clearing abnormally aggregated α-synuclein (αSyn). In preclinical Parkinson’s disease (PD) models, NK cell depletion worsens motor deficits and increases insoluble αSyn accumulation, suggesting a neuroprotective role. However, the therapeutic potential of NK cell transfer in modulating αSyn pathology and neurodegeneration remains unexplored.

**Methods:**

To assess the efficacy of NK cell therapy, we administered biweekly systemic injections of untouched NK cells isolated from B6C3H donor mice into 2-month-old presymptomatic homozygous M83 transgenic mice injected with human αSyn preformed fibrils. Neurological function was assessed via clasping behavior and clinical scoring. αSyn pathology and dopaminergic neurodegeneration were evaluated via immunohistochemistry. CyTOF-based immune profiling and multiplex ELISA were performed to characterize central and peripheral immune responses.

**Results:**

Adoptive NK cell transfers improved motor function and reduced αSyn pathology in a region- and dose-dependent manner, with significant reductions in phosphorylated-αSyn inclusions and tyrosine hydroxylase-positive neuronal loss in the substantia nigra. NK cell transfer modulated the CNS immune landscape by reducing CD11b^+^CD45^high^ and MHCII^+^ activated microglial, CD4⁺ T cells, and neutrophil infiltration, while promoting CD19^+^ B and CD8^+^ T cells. Similar immunomodulatory effects were observed in the periphery, including restoration of follicular B cells and reduced neutrophil frequencies. Mechanistically, αSyn exposure downregulated activating NK ligands and upregulated inhibitory receptor ligand mQa1b, along with p21 induction in microglia, suggesting a senescence-associated, immune-evasive phenotype that may contribute to reduced therapeutic efficacy at later disease stages.

**Conclusions:**

Our study provides direct evidence of NK cells exerting neuroprotective and immunomodulatory effects in a preclinical model of synucleinopathy. These findings support NK cell transfer as a novel therapeutic strategy for PD and related neurodegenerative disorders.

**Supplementary Information:**

The online version contains supplementary material available at 10.1186/s44477-025-00006-9.

## Background

Parkinson’s disease (PD) is the second most common neurodegenerative disease after Alzheimer’s disease [1]. It is characterized by the aggregation of misfolded α-synuclein (αSyn), leading to Lewy body and Lewy neurite formation, as well as the progressive dopaminergic neurodegeneration in the substantia nigra pars compacta (SNpc) [2, 3]. These pathological changes manifest as motor symptoms, including bradykinesia, tremor, rigidity, and postural instability [4]. While PD is primarily defined by its motor symptoms, non-motor symptoms such as cognitive decline, depression, gastrointestinal dysfunction, and sleep disturbances are increasingly recognized as major contributors to disease burden and reduced quality of life [5, 6].

Growing evidence suggests that neuroinflammation plays a critical role in PD progression. Elevated levels of pro-inflammatory cytokines including IL1β, IL2, IL6, IFNγ, and TNFα have been detected in the serum and cerebrospinal fluid (CSF) of PD patients [7], and they correlate with disease severity [8]. One potential driver of this inflammation is αSyn aggregation, which has been shown to hyperactivate microglia, leading to increased production of pro-inflammatory cytokines and reactive oxygen species, further exacerbating neuronal damage [9]. The immune system plays a dual role in PD, contributing to both disease progression and potential neuroprotection. Clinical and preclinical studies have reported an increased proportion of CD8^+^ T cells and a reduction in CD4^+^ T cells in the peripheral blood of PD patients [10]. Notably, CD4^+^ T cell depletion protected mice from dopaminergic neurodegeneration in a mouse model of PD [11]. Conversely, B cell depletion has been linked to greater dopaminergic neuron loss and worsened motor deficits, highlighting the complex role of adaptive immunity in PD [12].

NK cells are innate lymphocytes of growing interest in PD due to their dynamic roles in immune surveillance and emerging evidence of their involvement in neurodegenerative processes [13]. NK cells are traditionally recognized as rapid responders capable of eliminating "missing-self" cells through cytotoxic mechanisms, and they play critical roles in antimicrobial defense [14, 15], clearance of senescent cells [16], regulation of inflammation [17, 18], and modulation of adaptive immune responses [19, 20]. Recent transcriptomic studies have confirmed that NK cells reside within the brain under both homeostatic and pathological conditions [21, 22]. In PD patients, NK cell numbers are higher in the blood and their activity level is associated with disease severity [23–25]. In wild-type mice injected with αSyn PFF, NK cell frequency in the CNS increased compared to monomer (mono)-injected controls [26]. Our previous work further demonstrated that systemic depletion of NK cells exacerbates disease pathology in the αSyn preformed fibril (PFF) M83 transgenic (Tg) mouse model. NK cell-deficient mice exhibited significantly greater accumulation of αSyn pathology accompanied by elevated neuroinflammation [27], suggesting a protective role for NK cells in limiting both αSyn burden and inflammatory activation during synucleinopathy. Building on these findings, the current study investigates whether adoptive NK cell transfer can therapeutically modulate disease progression, reduce neuropathology, and restore immune balance in the M83 α-synucleinopathy model.

We previously demonstrated that systemic NK cell depletion in a preclinical PD mouse model exacerbated αSyn pathology in the striatum, SNpc, and brainstem, increased neuroinflammation and worsened motor deficits [28], supporting a protective role of NK cells in PD. These findings suggested that NK cells modulate neuroinflammation and facilitate the clearance of pathological protein aggregates [28]. However, it remains unclear whether NK cell enrichment can mitigate motor deficits and reduce αSyn pathology. To address this, we employed the (PFF-αSyn-injected M83 Tg mouse model, which recapitulates key features of αSynucleinopathies, including aggregate propagation and motor deficits. Using this model, we investigated whether systemic enrichment of NK cells modulates motor impairments and αSyn pathology. We assessed behavioral outcomes, synuclein burden, neurodegeneration, and neuroinflammatory responses, including immune cell profiling in the CNS and periphery using mass cytometry. NK cell transfer resulted in dose-dependent improvements in motor performance and reductions in neuroinflammation. These effects were accompanied by decreased αSyn accumulation in key brain regions, suggesting a potential modulatory role for NK cells in the progression of synucleinopathy. Together, these findings provide important insights into the therapeutic potential of NK cell-based interventions in Parkinson’s disease.

## Methods

### Mice

We used male and female M83 Tg mice (B6;C3-Tg(Prnp-SNCA*A53T)83Vle/J, homozygous, #004479 Jackson Laboratories) and B6C3F1/J mice (#100,010 Jackson Laboratories). Mice were maintained in a climate-controlled facility with a 12-h light/dark cycle. All research was performed in accordance with the NIH Guidelines for Animal Care and Use and approved by the Institutional Animal Care and Use Committee at the University of Georgia.

### Recombinant αSyn and preformed fibril preparation

Recombinant αSyn and fibrils were prepared as previously described [28]. Recombinant human αSyn was expressed in E. coli BL21(DE3)/RIL cells and purified to > 98% purity using size exclusion chromatography followed by Mono Q ion exchange chromatography, and further processed with high S cation exchange chromatography to reduce endotoxin, as previously described [29, 30]. Final endotoxin levels were confirmed to be below 0.5 EU/mg. To validate the absence of biologically active endotoxin, a QUANTI-Blue™ assay was performed to assess TLR4-mediated NF-κB activation using HEK-Blue™ TLR4 reporter cells. For fibril formation, purified αSyn monomers were incubated at 37 °C at a concentration of 5 mg/mL with constant shaking (1100 rpm) for 7 days. Prior to surgical injection or in vitro application, fibrils were sonicated using a high-intensity Cup Horn sonicator (Qsonica Q700, 30% amplitude for 30 min). Fibril morphology and seed size were verified by transmission electron microscopy (TEM) as previously published [28].

### Stereotaxic surgery

Homozygous M83 Tg mice (8-week-old, male and female) were anesthetized with ketamine/xylazine (100 mg/kg and 10 mg/kg) and placed in a stereotaxic frame (KOPF) equipped with ear bars. Using sterile technique, animals received a unilateral intrastriatal injection of either human PFF αSyn or mono αSyn (5 µg in 1 µL) into the right striatum. Stereotaxic coordinates relative to bregma were: anterior–posterior (AP) + 0.3 mm, medial–lateral (ML) + 2.3 mm, and dorsal–ventral (DV) − 3.5 mm from the dural surface. Injections were delivered at a rate of 0.2 μL/min using a 29-gauge Hamilton syringe. Postoperatively and for the following 3 days, animals received subcutaneous injections of analgesics and were monitored meticulously for signs of pain and discomfort.

### NK cell isolation and flow cytometry

Following anesthesia with isoflurane, spleens were harvested from donor mice and processed to isolate splenocytes. Spleens were mechanically minced and suspended in cold PBS. After centrifugation at 1500 rpm for 10 min at room temperature, red blood cells were lysed using ACK lysing buffer (Quality Biological, Catalog# 118–156-101). Following lysis, cells were washed once with PBS, counted, and resuspended in PBS containing 2% fetal bovine serum (FBS). NK cells were then isolated using the EasySep™ Mouse NK Cell Isolation Kit (Stemcell Technologies, Catalog# 19,855) following the manufacturer’s protocol. The resulting enriched NK cell population—negatively selected and untouched—was resuspended in sterile 0.9% sodium chloride (normal saline) for injection. NK cells were injected to recipient mice via intraperitoneal (i.p.) injection. To evaluate the purity and subset distribution of isolated NK cells, splenocytes or isolated NK cells were resuspended in FACS buffer and stained for 30 min at 4 °C with TruStain FcX™ anti-mouse CD16/32 antibody (BioLegend, Cat# 101,319) to block Fc receptors. Cells were subsequently incubated with fluorophore-conjugated antibodies against NK1.1 (Cat# 156,514), CD45 (Cat# 109,827), CD3 (Cat# 100,241), CD19 (Cat# 115,512), and CD11b (Cat# 101,208), all from BioLegend. Following staining, cells were washed twice in 200 µL of FACS buffer and analyzed using the Novocyte Quanteon flow cytometer. Data were analyzed using FlowJo™ software (BD Biosciences).

### Behavior assessment

To assess motor performance and disease progression, hind limb clasping behavior was evaluated in accordance with previously published protocols [31]. Mice were gently suspended by the base of the tail, and the position of the hind limbs was observed over a 30-s period. Mice were tested every other week. Each session included three independent trials spaced one hour apart, and the average score across the three trials was recorded as the final score for that time point. Clasping behavior was scored on a 0–3 scale: a score of 0 indicated normal hind limb extension with no retraction; a score of 1 was assigned if one hind limb was retracted for at least 50% of the observation period; a score of 2 was given if both hind limbs were partially retracted for at least 50% of the time; and a score of 3 represented full retraction of both hind limbs for at least 50% of the trial.

At the study endpoint, mice underwent a final clinical evaluation to quantify cumulative motor and neuropathological deficits. A clinical scoring system was used, adapted from previous studies [31], which ranged from 0 to 5. A score of 0 indicated a normal, healthy phenotype; 1 represented an unsteady gait; 2 reflected limb weakness or occasional tripping; 3 indicated severe limb weakness, though the animal was still mobile and able to access food; 4 described a state of paralysis or profound weakness where movement was minimal or absent; and 5 indicated that the mouse was moribund or deceased.

### Immunohistochemistry

Mice were euthanized and transcardially perfused with 0.1 M phosphate-buffered saline (PBS) followed by 4% paraformaldehyde (PFA). Brains were post-fixed in 4% PFA for 24 h and then cryoprotected in 30% sucrose. Coronal sections (30 µm thick) were prepared and stored in 0.1 M PBS containing 0.05% sodium azide until further use.

For brightfield immunohistochemistry, free-floating brain sections were first washed in 0.1 M phosphate-buffered saline (PBS) and quenched with 3% hydrogen peroxide to block endogenous peroxidase activity. Following additional washes, sections were incubated in a blocking solution containing 8% appropriate normal serum, 0.1% Triton X-100, and 0.1 M Tris-buffered saline (TBS-T) for 1 h at room temperature. Sections were then incubated overnight at 4 °C with primary antibodies against phosphorylated αSyn at Ser129 (p-αSyn) (Abcam, ab51253; 1:150,000) or tyrosine hydroxylase (TH) (Chemicon/Millipore, AB152; 1:2,500), diluted in 2% normal serum, 0.1 M TBS, and 50 µg/mL biotin (Vector Laboratories). To detect the insoluble form of p-αSyn, brain sections were incubated with proteinase K (PK; 10 µg/mL; MP Biomedicals, 183988) in 0.1 M TBS for 5 min at room temperature. The next day, sections were washed and incubated for 1 h at 4 °C with a biotinylated secondary antibody (Vector Laboratories) diluted in TBS-T. VectaStain® Elite ABC peroxidase kit (Vector Laboratories, PK-6100), and DAB kit (Vector Laboratories, SK-4100) were used according to the manufacturer’s protocol to develop horseradish peroxidase activity. Stained sections were mounted onto Plus™ coated glass slides, dehydrated and coverslipped using permanent mounting medium (Vector Laboratories, H-5700–60).

For immunofluorescence, free-floating brain sections underwent antigen retrieval by incubation in citrate buffer (0.1 M citric acid and 0.1 M tris-sodium citrate) at 60 °C overnight. Sections were then washed and quenched with 0.2 M glycine in PBS for 1 h. Permeabilization was performed using 0.3% Triton X-100 and 1% normal serum in 0.1 M TBS for 35 min at room temperature. Then, sections were blocked in 1% normal serum, followed by overnight incubation at 4 °C with primary antibodies: Iba1 (Synaptic Systems, 234 308, 1:500), and p-αSyn (Abcam, ab51253, 1:25,000) diluted in 1% normal serum, 0.1% Triton X-100, and 0.1 M TBS. The next day, sections were washed with TBS-T (0.05% Triton X-100) and incubated for 2 h at room temperature with corresponding Alexa Fluor® secondary antibodies. Sections were incubated in PBS containing Hoechst (Invitrogen, H3569; 1:10,000) for 5 min. After washes, sections were mounted with antifade medium (Invitrogen, P36930).

### Microscopy and quantitative image analysis

Brightfield images for p-αSyn and TH were acquired using a Keyence BZ-X800 microscope. Images were taken at 20X magnification from the cortex, striatum, SN, brainstem were used for the analysis. Immunofluorescent images for Iba-1 and p-αSyn were acquired using a Zeiss AxioImager M2 microscope. For each SN, z-stack images were collected at 1 μm intervals across the thickness of the Sect. (30 μm) using a 40X objective. Z-stacks were processed into maximum-intensity projections for analysis. NIHImageJ1.53 software (National Institute of Health) was used for quantification on the lesioned side. The total area of immuno-stained area with threshold parameters that excluded the background staining was detected. The average total area of the immuno-reactive area on each ROI for all images was plotted for analysis.

### Stereological analysis

Unbiased stereological estimation of TH-positive (TH⁺) dopaminergic neurons in the SNpc was performed. SNpc was delineated using a 5X objective, and dopaminergic (DA) neurons were counted under a 40X objective using a Zeiss AxioImager M2 microscope. Serial coronal brain Sects. (30 μm thickness; mounted thickness ~ 22 μm) were placed six per slide. Every other slide was stained with TH and bisbenzimide for neuron identification. Stereological analysis was conducted using a systematic random sampling approach with counting frames (50 × 50 μm) overlaid on a sampling grid (190 × 130 μm). Cells were counted within an 18 μm optical dissector, using 2 μm guard zones at the top and bottom of each section to avoid edge artifacts.

### Western blot analysis of spinal cord tissue

Spinal cord regions of interest were dissected from PFA-fixed tissue and transferred into 1.5 mL microcentrifuge tubes containing 150 µL of lysis buffer (300 mM Tris–HCl, pH 8.0, 2% SDS, 1% Triton X-100) supplemented with protease inhibitor cocktail (Roche, 04693116001). Tissues were sonicated at 30% amplitude using a cycle of 5 s on and 5 s off, repeated three times. Lysates were incubated at 100 °C for 30 min followed by 80 °C for 2 h to enhance protein solubilization. After centrifugation, supernatants were collected for SDS-PAGE analysis. Protein samples were separated on 4–20% TGX Stain-Free gels (Bio-Rad) and imaged using the ChemiDoc MP system (Bio-Rad). Proteins were transferred to membranes and probed with anti-p-αSyn (Abcam, ab51253) and anti-αSyn (BD Bioscience, 610787), followed by HRP-conjugated secondary antibodies (1:2000; Jackson ImmunoResearch). Immunoreactive bands were detected using SuperSignal West Pico chemiluminescent substrate (Thermo Fisher Scientific) and quantified using Image Lab software (Bio-Rad).

### Isolation of brain mononuclear immune cells

Mononuclear cells were isolated from mouse brains as previously described [26, 32]. Briefly, mice were deeply anesthetized and perfused with PBS. Brains were extracted, minced in 3 mL of ice-cold 1 × HBSS, and enzymatically digested in 3 mL of 2X dissociation medium containing DNase I (1 µL/mL; Invitrogen), Dispase II (1.2 U/mL; Roche), and Papain (1 mg/mL; Sigma-Aldrich) in DMEM/F12. Tissues were triturated using polished Pasteur pipettes of decreasing diameter, resuspended in 4 mL of 37% isotonic Percoll (Sigma), and subjected to density gradient centrifugation using a 30:37:70 Percoll step gradient. Gradients were centrifuged at 500 × g for 30 min, and mononuclear cells were collected from the 37–70% interface. Cells were washed twice in HBSS, counted, and processed for CyTOF Immunostaining.

### Mass cytometry

Single-cell suspensions were prepared from mouse spleen and brain tissue following PBS perfusion. After tissue dissociation and isolation, brain cells were pooled (n = 5–6) due to the low yield of immune cells from individual brains. In contrast, splenocytes were processed individually. Cells were washed and resuspended in Maxpar Cell Staining Buffer (CSB, Standard BioTools/Fluidigm, 201068). Cells were aliquoted and blocked Fc receptor by adding 5 µL of Mouse TruStain FcX (BioLegend, 422302). Meanwhile, surface marker cocktails containing 33 metal-conjugated antibodies (Maxpar OnDemand™ Mouse Immune Profiling Panel Kit, 92-000-001*)* and Cell-ID Intercalator-103Rh (2 µM final; Fluidigm) were prepared and incubated with cells for a 30-min at room temperature. For fixation and DNA intercalation, cells were resuspended in 400 µL PBS and mixed with 400 µL of 2X Fix-IR solution containing isotonic paraformaldehyde, 2% saponin, Cell-ID Intercalator-Ir (125 µM; Fluidigm, S00093). Samples were incubated at room temperature for 30 min, followed by two washes with CSB at 800 × g. Samples were resuspended in deionized water containing EQ™ Four Element Calibration Beads (Fluidigm) and acquired using a CyTOF XT mass cytometer. Brain immune profiles were analyzed using FlowJo software (version 10), and splenocyte populations were analyzed using Maxpar® Pathsetter™ software (version 3.0).

### Primary cultures

Primary mixed neuron-glia cultures were prepared from the hippocampi of postnatal day 0–2 mice. Hippocampi were dissected, meninges removed under a stereomicroscope, and tissue minced and enzymatically digested in a solution containing 20 U/mL DNase I, 1 mg/mL papain, and 1.2 U/mL dispase II for 15 min at 37 °C. The reaction was quenched with fetal bovine serum (FBS), and cells were pelleted by centrifugation. Cells were resuspended in plating medium (Neurobasal A supplemented with 5% FBS, 2 mM Glutamax, and 10 μg/mL gentamycin), mechanically triturated using fire-polished glass pipettes, and filtered through a 40 μm cell strainer. Cells were seeded at 100,000 cells per 80 μL onto poly-D-lysine–coated coverslips (100 μg/mL) in 24-well plates. The next day, half of the medium was replaced with inhibitor medium containing 2 μg/mL aphidicolin to limit glial overgrowth. From that point, half-medium changes with culture medium (Neurobasal A + 1X B27 + 2 mM Glutamax) were performed every 4 days. On day 7, cultures were treated with sonicated αSyn PFF (1 or 1.5 μg/mL final concentration) or PBS. PFFs were prepared by mixing 10 μL of stock PFFs (4.38 mg/mL) with 10 μL PBS, followed by sonication. On day 14, primary NK cells were isolated from mouse spleen and added to selected wells at 10,000 cells/well (n = 6 per condition). Supernatants were collected after 24 h for cytokine analysis using Meso Scale Discovery (MSD) assays, and cells were fixed on day 15 for immunostaining.

Primary microglial and astrocyte cultures were prepared from the cortices of postnatal day 3–6 mouse pups (n = 6–8). Cortical tissue was isolated, minced, and enzymatically dissociated in 0.25% Trypsin–EDTA at 37 °C for 20 min with agitation every 5 min. Trypsin was neutralized with complete medium consisting of DMEM/F12 supplemented with 10% heat-inactivated fetal bovine serum (FBS; Sigma), 1% penicillin–streptomycin, and 1% L-glutamine (Sigma). The resulting cell suspension was plated and maintained at 37 °C in 5% CO_2_. For astrocyte cultures, cells were used after 4 days in vitro. For microglial isolation, mixed glial cultures were maintained for 12 days, after which microglia were harvested by mechanical agitation. Isolated microglia were replated in DMEM/F12 supplemented with 10% heat-inactivated FBS.

Bone marrow-derived macrophages (BMDMs) were generated as previously described [33]. Briefly, bone marrow cells were isolated from the femurs and tibias of mice and cultured in DMEM/F12 supplemented with 10% fetal bovine serum (FBS) and 20% L929 cell-conditioned medium, which serves as a source of macrophage colony-stimulating factor (M-CSF), for 7 days. To reduce M-CSF signaling and minimize macrophage activation prior to experimentation, BMDMs were maintained in medium without L929-conditioned medium for an additional 2 days.

### Immunocytochemistry

Primary neuron glia culture was incubated overnight at 4 °C with anti-MAP2 (Invitrogen; PA1-16751, 1:1000) and anti-p-αSyn (Abcam; ab51253, 1:200) antibodies. Following primary incubation, cells were washed and incubated for 2 h at room temperature (RT) with Alexa Fluor 594- and Alexa Fluor 488-conjugated secondary antibodies (Invitrogen; 1:1000). Coverslips were mounted using Fluoroshield Mounting Medium containing DAPI (Abcam, ab104139). All fluorescent images were acquired using a Nikon A1R confocal microscope mounted on a Nikon Eclipse Ti-E inverted microscope platform.

### Multiplex ELISA

Supernatants from primary cultures or serum samples were collected for cytokines and chemokines analysis. The Mouse Proinflammatory Panel 1 (Meso-Sacle Discovery, Gaithersburg, MD) was used to quantify IFNγ, IL-10, IL-12p70, IL-1, IL-2, IL-4, IL-5, IL-6, KC/GRO, and TNFα levels, following the manufacturer’s instructions.

### Quantitative real-time RT-PCR (qPCR)

Total RNA was extracted from cultured cells using the RNeasy Mini Kit (Qiagen) and treated with DNase I to eliminate genomic DNA contamination. First-strand cDNA synthesis was performed using SuperScript II RNase H⁻ reverse transcriptase (Invitrogen). qPCR was conducted using SYBR Green Master Mix in a 384-well format on a QuantStudio 6 Real-Time PCR System (Thermo Fisher Scientific). Primer pairs were synthesized by Integrated DNA Technologies (IDT, Coralville, IA). The following primer sequences were used: *Mult1* (Forward: 5′-ACT GGC CAC ACA CCT CAG C-3′, Reverse: 5′-TTC ACA TAG TGC AGG AGA CTA ACA CA-3′); *Qa-1a* (Forward: 5′-TCT GTG AGG CAA AGT CAG TC-3′, Reverse: 5′-GCG GTA TTT CCA CAC TGC CA-3′); *Qa-1b* (Forward: 5′-CAC CAC AGC TCC AAG GAT GAT-3′, Reverse: 5′-CCT GGA CCG CGA ATG ACA TA-3′); *Rae-1* (Forward: 5′-AGA TAT GAA GAT GAG TCC CAC AGA GAT A-3′, Reverse: 5′-ATC AAC TTC CCC GCT TCC A-3′); *H60* (Forward: 5′-CCA GTA TGG TCC CCA GAT AGC T-3′, Reverse: 5′-GAG CCA CCA GCA AGA GCA A-3′); *p16* (Forward: 5′-TGT TGA GGC TAG AGA GGA TCT TG-3′, Reverse: 5′-CGA ATC TGC ACC GTA GTT GAG C-3′); and *p21* (Forward: 5′-TCG CTG TCT TGC ACT CTG GTG T-3′, Reverse: 5′-CCA ATC TGC GCT TGG AGT GAT AG-3′). All reactions were run in triplicate, and relative expression was calculated using the ΔΔCt method, normalized to housekeeping genes. Each reaction was run in triplicate, and data are presented as mean ± SEM.

### Statistical analyses

Statistical analyses were conducted using GraphPad Prism 10 software. Data are presented as mean ± SEM. Comparisons between treatment groups were performed using two-way ANOVA followed by Tukey’s multiple comparisons test or Fisher’s LSD post-hoc test. Comparisons within groups were analyzed using one-way ANOVA or Student’s unpaired t-test. A p-value of < 0.05 was considered statistically significant. Correlation analyses were performed using GraphPad Prism 10 software. The total number of TH^+^ neurons, the total Iba-1^+^ area or the total p-Ser129 αSyn^+^ area was used as a variable. Pearson’s correlation coefficients (r) were calculated to assess linear associations between variables. A significance threshold of p < 0.05 was applied. The strength and direction of the correlation were interpreted based on the r value, where + 1 indicates a perfect positive correlation, 0 means no correlation, and −1 indicates a perfect negative correlation.

## Results

### Systemic NK transfer leads to significant increases of NK cells in the spleen and blood of recipient mice

To evaluate the effects of NK cell transfer therapy in a mouse model of neurodegenerative disease, we stereotaxically injected 2-month-old homozygous M83 Tg mice (both males and females) with 5 μg of human αSyn PFF or mono αSyn into the striatum. To preserve NK cell phenotype and function, untouched NK cells were isolated from the spleens of background-matched B6C3F1/J mice (2–4 months old) using a negative selection approach (EasySep™ Mouse NK Cell Isolation Kit). NK subset analysis based on CD11b and CD27 markers revealed that the majority of enriched NK cells exhibited CD11b⁺CD27⁻ and CD11b⁺CD27⁺ phenotypes **(**Supplementary Fig. S1**)**.

**Fig. 1:**
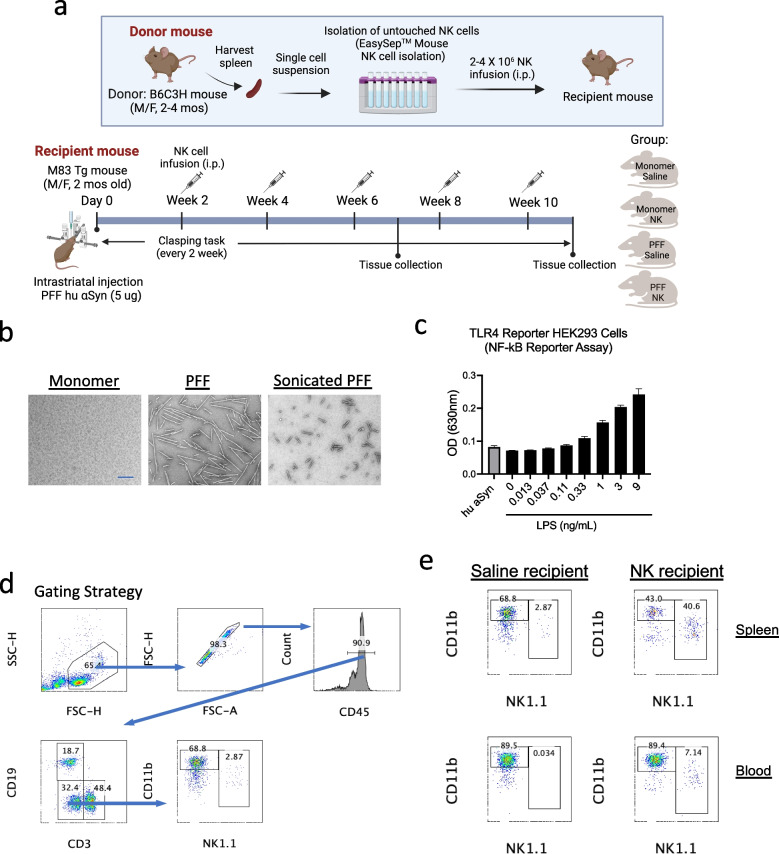
Systemic NK cell transfer leads to significant increases of NK cells in the spleen and blood of recipient mice. (**a**) Schematic of NK cell transfer experimental design. Untouched NK cells were isolated from the spleens of background-matched B6C3H donor mice (male and female, 2–4 mos old) using negative selection (EasySep™ Mouse NK Cell Isolation Kit). Single-cell suspensions of enriched NK cells (2 or 4 × 10⁶ cells) were administered intraperitoneally (i.p.) into recipient M83 Tg mice (homozygous, male and female, 2-mos old) that had been previously stereotaxically injected with 5 μg of human αSyn (hu αSyn) PFF or mono into the striatum. NK cell transfers were performed biweekly, starting at 2 weeks post-injection. Hindlimb clasping task was performed every two weeks. Tissues were collected at 7- and 11 weeks post-injection to evaluate αSyn pathology, neurodegeneration, and immune responses. **(b)** Representative images of αSyn mono, PFF, and sonicated PFF under TEM microscopy. Scale bar = 100 nm. **(c)** TLR4 reporter assay using HEK293 cells demonstrates that hu aSyn (5 ug) shows minimal activation, while lipopolysaccharide (LPS) induces dose-dependent activation of NF-κB signaling. **(d)** Gating strategy for flow cytometry. **(e)** Flow cytometry analysis of NK cells in spleen and blood samples from recipient mice were performed 3 days after i.p. injection

Given the turnover rate of circulating human NK cells and adoptively transferred murine NK cells (~ 7–14 days) [34–36], NK cells were administered intraperitoneally (i.p.) at two-week intervals to maintain consistent immunological pressure throughout disease progression. The dosing regimen (2 or 4 million cells per injection) and transfer timeline were adapted from previously validated protocols [35, 37]. Successful engraftment of NK1.1⁺ cells in recipient mice confirmed the efficacy of this systemic delivery strategy. NK1.1⁺ populations were substantially elevated in both the spleen and circulating blood of NK-treated mice compared to saline-treated controls **(**Fig. 1e**)**, demonstrating that intraperitoneally administered NK cells can persist and expand in vivo. This engraftment established a robust immunological foundation for further evaluating NK cell–mediated modulation of αSyn pathology, neuroinflammation, and neuronal survival. Motor function was assessed biweekly following NK cell administration. Tissues were harvested at weeks 7 and 11 post-injections for analysis of αSyn pathology, neuroinflammation, and therapeutic responses **(**Fig. 1a**)**. The conformations of αSyn species (mono, PFF, and sonicated PFF) were validated by transmission electron microscopy **(**Fig. 1b**)**, and TLR4 reporter assays using HEK293 cells confirmed minimal biological activity of the αSyn preparations **(**Fig. 1c**)**.

### NK cell transfer mitigates motor deficits, DA neurodegeneration and insoluble p- αSyn burden in the brain 

**Fig. 2:**
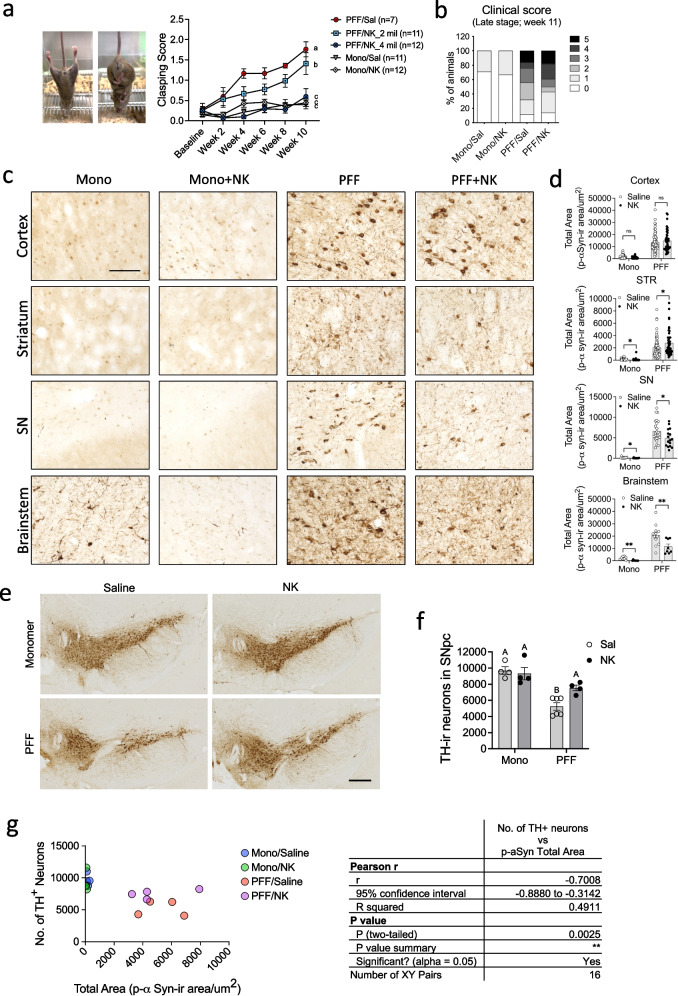
NK cell transfer mitigates motor deficits, DA neurodegeneration and insoluble p-αSyn burden in the brain. M83 Tg mice were unilaterally injected with either mono or PFF αSyn into the striatum and treated biweekly with saline or NK cells (4 ×10⁶ cells per dose, i.p.). Mice were analyzed at 11 weeks post-injection. (**a**) Representative images show clasping scores of 0 (left) and 3 (right). Graph shows longitudinal clasping scores. Data analyzed by two-way ANOVA with Tukey’s post hoc test (*n* = 7–12 mice/group). Different letters indicate statistically significant group differences (*p* < 0.05). Error bars represent mean ± SEM. (**b**) Clinical symptoms were assessed at endpoint (11 weeks post-injection) as detailed in Methods. (**c**) Representative immunohistochemical images of PK-resistant p-αSyn pathology in cortex, striatum, SN, and brainstem. Scale bar = 100 µm. (**d**) Graphs show quantification of total PK-resistant p-αSyn^+^ area per region of interest (n = 4 mice/group). Statistical analysis by two-way ANOVA with Fisher’s LSD post hoc test. *p < 0.05, **p < 0.01. Error bars = mean ± SEM. (**e**) Representative images of TH^+^ neurons in the SNpc. Scale bar = 200 µm. (**f**) Quantification of TH^+^ neurons using unbiased stereological analysis. Data analyzed by two-way ANOVA with Fisher’s LSD post hoc test. Different letters indicate statistically significant group differences. Error bars represent mean ± SEM. (**g**) Correlation between TH^+^ neuron counts and the total p-αSyn^+^ area across groups. Each data point represents an individual animal. Pearson’s correlation coefficient (r) is shown; *p* < 0.05 was considered significant

To determine whether NK cell transfer modulates disease progression in synucleinopathy, we assessed motor function and neuropathology in M83 Tg mice injected with αSyn PFF (PFF) or mono αSyn (Mono). Hind limb clasping scores were recorded biweekly. Mice treated with PFF and saline exhibited the most severe motor impairments, with scores approaching 2.0 by Week 10. In contrast, PFF-injected mice treated with NK cells (PFF + NK) showed a dose-dependent protective effect, with the 4 million NK cell group demonstrating the greatest improvement **(**Fig. 2a**)**. Clinical scores were assessed at the endpoint (11 weeks) as the homozygous M83 Tg mice injected with αSyn PFF typically display symptom onset around 10 weeks post-injection, followed by a rapid clinical decline within a few days, as previously observed [28]. Due to this late onset and fast progression, scores were recorded on the day of sacrifice. Clinical scores revealed that over 50% of PFF mice developed severe symptoms (scores 3–5). Interestingly, PFF + NK mice exhibited similar levels of clinical severity at this late stage, indicating limited therapeutic benefit of NK cells during advanced disease **(**Fig. 2b**).**

We next evaluated αSyn pathology by performing immunohistochemistry for proteinase K (PK)-resistant p-αSyn. Our data revealed extensive αSyn inclusions in the cortex, striatum, substantia nigra (SN), and brainstem of PFF mice at 11 weeks **(**Fig. 2c and d**)**. NK cell transfer (4 million per dose) significantly reduced αSyn pathology in the SN (*p < 0.05) and brainstem (**p < 0.01) compared to the PFF group. A modest increase in striatal pathology was observed. Notably, in the mono-injected NK-treated group (Mono + NK), high-dose NK cell transfer reduced αSyn pathology in the striatum (*p < 0.05), SN (*p < 0.05), and brainstem (**p < 0.01) compared to mono-injected saline-treated controls (Mono). The cortex showed a reduction trend, but it did not reach statistical significance. We also observed that low-dose NK cell transfer (2 milion per dose) led to significant reductions in αSyn pathology, particularly in striatal (**p < 0.01) and SN (*p < 0.05). No significant differences in αSyn pathology were observed in the cortex or brainstem across both PFF and mono-injected groups. (Supplementary Fig. 2).

Stereological quantification of tyrosine hydroxylase (TH)-positive neurons in the SNpc revealed that PFF injection caused an approximately 47% reduction in dopaminergic neurons compared to Mono injection (p < 0.05) **(**Fig. 2e and f**)**. PFF + NK mice exhibited significantly higher TH⁺ neuron counts compared to the PFF group, indicating NK cell-mediated neuroprotection. To further examine whether the αSyn pathology is associated with DA neuron degeneration, we performed correlation analyses among TH^+^ neuronal counts and αSyn pathology. At 11 weeks p.i., the number of DA neurons in SN was negatively correlated with pathological αSyn burden (total p-αSyn^+^ area vs. number of DA neurons: r = −0.7, *p* < 0.01) **(**Fig. 2g**)**.

### NK cell transfer reduces p-αSyn burden in spinal cord and microglia activation correlates with p-αSyn burden in SN at mid-stage disease

**Fig. 3:**
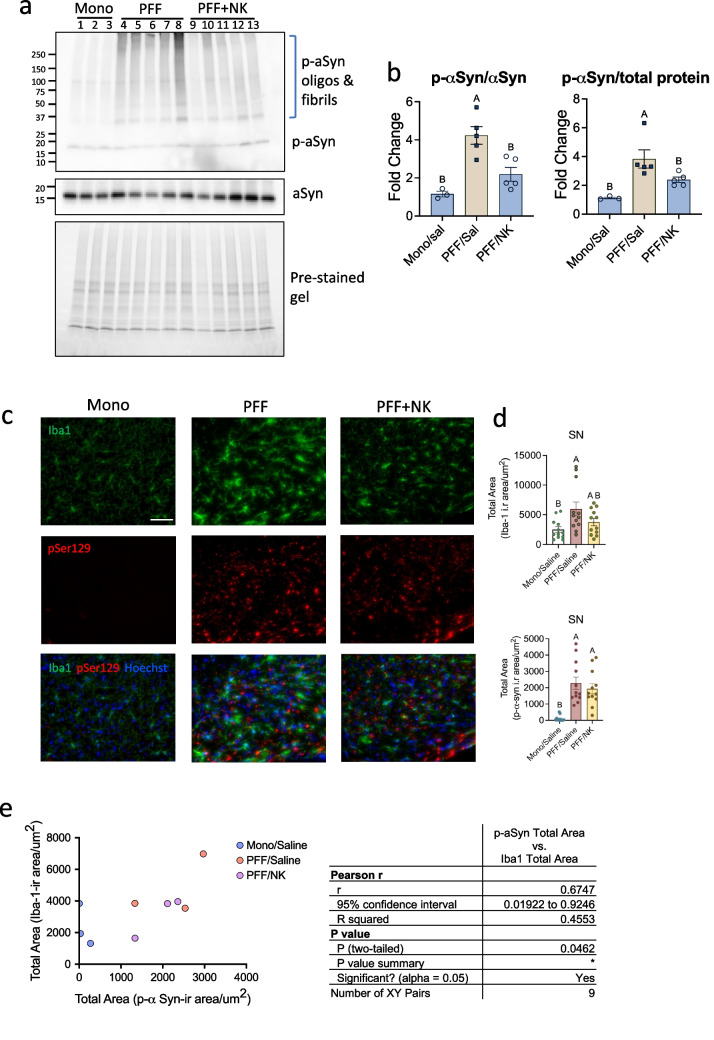
NK cell transfer reduces p-αSyn burden in spinal cord and microglia activation correlates with p-αSyn burden in SN at mid-stage disease. M83 Tg mice were unilaterally injected into the striatum with either mono αSyn (Mono) or αSyn PFF. Mice treated biweekly with saline or NK cells (4 ⅹ 10⁶ cells per dose, i.p.). At 7 weeks p.i., mice were sacrificed and spinal cord and brain tissues were analyzed. (**a**) A representative immunoblot of p-αSyn and αSyn in the lumbar spinal cord. Loading lanes: 1–3, Mono + saline (Mono); 4–8, PFF + saline (PFF); 9–13, PFF + NK. (**b**) Quantification of p-αSyn normalized to total protein and total αSyn levels. Data represent mean ± SEM (*n* = 3–5 mice/group). One-way ANOVA was used for statistical analysis. Distinct letters indicate significant differences (*p* < 0.05). (**c**) Representative images of the substantia nigra stained for Iba-1 and p-αSyn. Scale bar = 50 µm. (**d**) Graphs show quantification of total p-αSyn^+^ area or total Iba1^+^ area per region of interest. One-way ANOVA was used for statistical analysis. Distinct letters indicate significant differences (*p* < 0.05). (**e**) Correlation analysis between Iba-1^+^ area and p-αSyn^+^ area. Data points represent individual mice. Pearson’s correlation coefficient (r) was calculated, and correlations with p < 0.05 were considered significant

At week 7 (mid-stage disease), a time point of peak behavioral improvement following NK cell treatment, we performed quantitative immunoblotting of p- αSyn across the lumbar, thoracic, and cervical spinal cord. PFF + NK mice exhibited a ~ 60% reduction in αSyn pathology compared to PFF, restoring levels to those of Mono controls in the lumbar spinal cord **(**Fig. 3a and b**)**. A similar, though less pronounced, reduction was observed in the thoracic spinal cord (Supplementary Fig. 3a and c). In the cervical spinal cord, a reduction in αSyn levels was also noted in PFF + NK mice; however, this difference did not reach statistical significance (Supplementary Fig. 3b and d). These results suggest a rostro-caudal gradient in NK cell efficacy, with maximal benefit in the lumbar spinal cord and limited effect in the cervical region at this time point.

We next conducted immunofluorescent analyses of αSyn pathology and microglial burden in the SN using p-αSyn and total Iba-1^+^area. Although no significant differences were observed, a decreasing trend in microglial burden (total Iba-1^+^ area) was detected in the PFF + NK group compared to PFF group, while no significant changes in αSyn burden were observed between PFF groups at 7 weeks p.i. **(**Fig. 3c and d**)**. Importantly, at 7 weeks p.i., p-αSyn burden was positively correlated with microglial burden (total Iba-1^+^ area) in the SN (r = 0.6747, *p* < 0.05) **(**Fig. 3e**)**. These findings suggest that PFF αSyn injection induces microglial activation in the SN.

### CyTOF-based profiling reveals NK cell transfer modulates brain-infiltrating immune populations and reduces inflammation at mid-stage disease

**Fig. 4:**
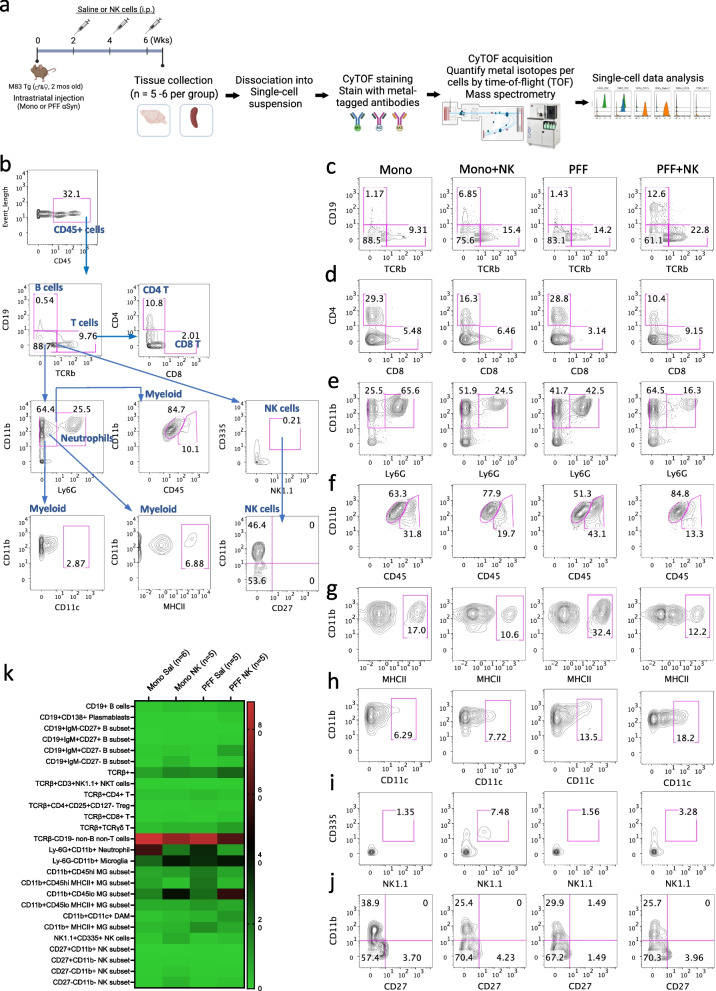
NK cell transfer reduces neuroinflammation and peripheral cell infiltration in the CNS in the αSyn PFF M83 Tg mice. (**a**) Schematic of the experimental workflow. M83 Tg mice received unilateral intrastriatal injections of mono or αSyn PFF and were treated biweekly with saline or NK cells (4 × 10^6^ cells per dose). At 7 weeks post-injection, mice were perfused with PBS to remove circulating blood cells, brains were harvested, processed into single-cell suspensions, pooled, stained with metal-tagged antibodies, and analyzed by CyTOF mass cytometry. (**b**) Gating strategy for identification of major immune cell populations, including CD45⁺ leukocytes, B cells, T cells (CD4⁺, CD8⁺), myeloid cells and NK cells in saline injected non-surgical baseline control mice (pooled, n = 5). **(c–j)** Representative contour plots showing frequencies of immune subsets across treatment groups (*n* = 5–6 mice per group): B cells (**c**), T cells (**d**), neutrophils (**e**), myeloid cells (**f, g, h**), and NK cells (**i, j**). Experimental groups include Mono + saline (Mono), Mono + NK, PFF + saline (PFF), and PFF + NK. (**k**) Heatmap summarizing the relative abundance of immune cell subsets in each group

To investigate NK cell-mediated immune changes in the CNS and periphery, we performed mass cytometry (CyTOF) using metal-tagged antibodies to profile immune cell populations in the brain at 7 weeks post-injection—a time point selected based on peak behavioral improvement following NK cell treatment. To ensure analysis of CNS-resident and infiltrating immune cells, mice were perfused with PBS prior to tissue collection to remove circulating blood cells **(**Fig. 4a**)**.

In the Mono control group, B cells accounted for 1.17% of the total brain immune cells, while T cells comprised 9.31%. NK cell transfer in mono-injected mice (Mono + NK) increased B cell frequency nearly six-fold (6.85%) and elevated T cells to 15.4%. In the PFF group, B cell levels remained low (1.43%), but T cells rose to 14.2%. Notably, NK cell treatment in PFF-injected mice (PFF + NK) led to a striking expansion of CD19⁺ B cells to 12.6%-a nearly ninefold increase compared to PFF controls **(**Fig. 4c**)**. This pronounced B cell expansion suggests that NK cells may promote B cell–mediated immune responses during synucleinopathy.

T cell subset analysis revealed additional NK cell–driven changes. In Mono mice, CD4⁺ T cells were dominant (29.3%) over CD8⁺ T cells (5.48%). NK cell transfer (Mono + NK) decreased CD4⁺ T cells to 16.3% and increased CD8⁺ T cells to 6.46%. In the PFF group, CD4⁺ T cells remained elevated (28.8%), while CD8⁺ T cells declined to 3.14%, suggesting limited recruitment of cytotoxic T cells. Importantly, the PFF + NK group reversed this pattern, reducing CD4⁺ T cells to 10.4% (a 64% reduction vs. PFF), and increasing CD8⁺ T cells to 9.15% **(**Fig. 4d**)**. In addition, saline-injected control mice **(**Fig. 4b**)** exhibited low baseline frequencies of CD4⁺ T cells (10.8%) and CD8⁺ T cells (2.01%), raising the possibility that mono αSyn may alter CNS immune composition.

We next analyzed CD11b and CD45 expression within the non-B non-T and CD11⁺ myeloid population. We found that NK cell transfer significantly reshaped the myeloid landscape. Saline-injected non-surgery baseline control mice shown in the gating strategy **(**Fig. 4b**)** showed 25.5% neutrophils and 64.4% myeloid cells, reinforcing the notion that mono αSyn can modulate immune homeostasis. In PFF mice, NK cell transfer (PFF + NK) resulted in the most pronounced effect: reducing neutrophils to 16.3%, and increasing CD11b⁺Ly6G⁻ cells to 64.5% similar to the levels observed in the baseline control **(**Fig. 4e**).**

We also analyzed CD11b and CD45 expression within the non-B non-T and Ly6G- myeloid population, representing primarily microglia and infiltrated macrophages. In Mono mice, 63.3% of cells were CD11b⁺CD45^low^ (homeostatic microglia), and 31.8% were CD11b⁺CD45^high^ (activated microglia/infiltrated macrophages). PFF injection (PFF) shifted this ratio, decreasing CD45^low^ cells to 51.3% and increasing CD45^high^ cells to 43.1% **(**Fig. 4f**)**. NK cell transfer (PFF + NK) reversed this pattern, restoring CD45^low^ cells to 84.8% and reducing CD45^high^ cells to 13.3%—a 69% reduction compared to PFF. MHCII expression, a marker of myeloid activation, similarly varied across the groups. PFF mice showed the highest MHCII⁺ myeloid cell frequency (32.4%), nearly double that of Mono (17.0%). NK cell treatment suppressed MHCII expression in both mono- and PFF-injected mice, with PFF + NK mice showing a 62% reduction in MHCII⁺ cells (12.2%) compared to PFF **(**Fig. 4g**)**. In contrast, CD11c expression, related to disease-associated microglia/macrophages (DAMs), was lowest in Mono (6.29%) but increased with PFF injection (13.5%). Interestingly, NK treatment further elevated CD11c⁺ cells in PFF + NK mice to 18.2%, a 35% increase compared to PFF, suggesting that NK therapy may promote a beneficial DAM-like phenotype during αSyn pathology (Fig. 4h). For baseline comparison, saline-injected non-surgery control mice shown in the gating strategy exhibited 84.7% of CD11b⁺CD45^low^, 10.1% of CD11b⁺CD45^high^ cells, 6.88% of MHCII⁺ myeloid and 2.87% of CD11c⁺ cells **(**Fig. 4b**)**, further supporting the idea that mono αSyn subtly disrupts CNS immune homeostasis. Importantly, NK cell transfer in both PFF- and mono-injected mice restored the homeostatic immune profile to baseline level.

Finally, we assessed NK cell presence and phenotype post-transfer. CD335⁺ (NKp46⁺) NK cells were low in Mono (1.35%) and PFF (1.56%) but significantly increased following NK treatment in both Mono + NK (7.48%) and PFF + NK (3.28%) mice **(**Fig. 4i**)**. Despite successful engraftment, levels were lower in the PFF context, suggesting reduced persistence or recruitment. NK-treated groups showed high proportions of immature CD11b⁻CD27⁻ NK cells (~ 70%) and a reduction in mature CD11b⁺CD27⁻ subsets. Terminally differentiated CD11b⁺CD27⁺ NK cells were nearly absent across all groups **(**Fig. 4j**)**.

A heatmap summarizing immune subset abundance **(**Fig. 4k**)** highlights the PFF + NK group’s distinct immune profile: elevated CD19⁺ B and CD8⁺ T cells, reduced CD4⁺ T cells and neutrophils, decreased activated microglia, and increased NK cells. Together, these changes suggest a coordinated, potentially neuroprotective immune response facilitated by NK cell therapy in synucleinopathy.

### NK cells directly modulate neuroinflammatory responses in αSyn-challenged primary neuron-glia cultures

**Fig. 5:**
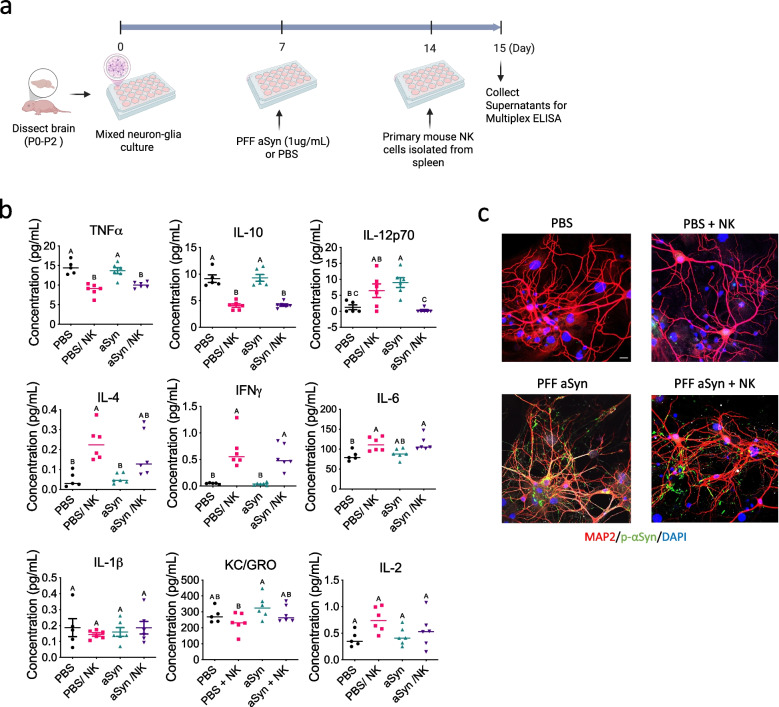
NK cells directly modulate neuroinflammatory responses in αSyn-challenged primary neuron-glia cultures. (**a**) Schematic of the mixed neuron-glia and NK cell co-culture experiment. Brains from postnatal day 0–2 (P0–P2) mice were dissected to establish mixed neuron-glia cultures. On Day 7, cultures were treated with either sonicated αSyn PFF (1 μg/mL) or PBS. On Day 14, NK cells isolated from mouse spleens were added to the cultures. Supernatants were collected on Day 15 for multiplex ELISA analysis of cytokine and chemokine profiles. (**b**) Quantification of cytokine levels (pg/mL) for TNFα, IL-10, IL-12p70, IL-4, IFNγ, IL-6, IL-1β, KC/GRO, and IL-2 across four experimental conditions. Data are presented as individual values with mean ± SEM. Statistical analysis was performed using one-way ANOVA with Tukey’s post hoc test. Groups labeled with different letters (A, B, C) are statistically different at p < 0.05. (**c**) Representative immunofluorescence images of neuron-glia cultures under the different treatment conditions. Red: neuronal marker; Green: glial marker; Blue: nuclear stain (DAPI). Scale bar: 50 μm

Given the systemic immunomodulatory effects observed with NK cell transfer in vivo, we next investigated whether NK cells could directly modulate immune responses in neuron-glia culture under αSyn-induced stress. Mixed neuron-glia cultures were established from postnatal day 0–2 mouse hippocampi. On day 7 in vitro, cultures were treated with either PBS or sonicated αSyn (αSyn PFFs; 1 μg/mL). On day 14, primary NK cells, isolated from mouse spleens, were added to the cultures. Supernatants were collected 24 h later for multiplex cytokine analysis **(**Fig. 5a**).** Co-culture of NK cells with αSyn-treated neuron-glia cultures induced measurable changes in the inflammatory milieu, with distinct cytokine signatures emerging across treatment conditions **(**Fig. 5b**).** TNFα levels were comparable between PBS and αSyn-treated cultures, indicating that αSyn did not further elevate TNFα beyond baseline. However, NK cell addition significantly reduced TNFα in both PBS and αSyn-treated cultures (~ 8–10 pg/mL), demonstrating NK-mediated suppression of both basal and αSyn-induced TNFα production **(**Fig. 5b**)**. IL-10 levels remained elevated (~ 8–13 pg/mL) following both PBS and PFF treatments, suggesting preserved anti-inflammatory tone. NK cell co-treatment led to a reduction in IL-10 levels across both conditions, indicating NK cell–mediated downregulation of IL-10 expression **(**Fig. 5b**)**. IL-12p70 exhibited context-dependent modulation. While low in PBS-only cultures, NK treatment modestly increased IL-12p70 under baseline conditions. αSyn exposure significantly upregulated IL-12p70, and NK cells reduced this elevation back to baseline, indicating suppression of pathology-driven IL-12p70 induction **(**Fig. 5b**)**.

IL-4 levels were low in PBS cultures (~ 0.05 pg/mL) but increased with NK co-treatment (~ 0.2 pg/mL), suggesting NK cell–driven IL-4 production in healthy conditions. In contrast, this NK cell–driven IL-4 induction was lost in αSyn-treated cultures **(**Fig. 5b**)**. IL-6 levels were significantly elevated in PBS/NK and αSyn/NK groups (~ 100–150 pg/mL) compared to PBS-only (~ 60–90 pg/mL), indicating that NK cell transfer enhances IL-6 production regardless of αSyn challenge **(**Fig. 5b**)**. In contrast, PFF alone did not significantly increase IL-6 compared to PBS, suggesting that the observed elevation is specifically driven by NK cell presence rather than αSyn pathology alone. This implies that NK cells may promote IL-6 production as part of a broader immunomodulatory response in both healthy and pathological conditions **(**Fig. 5b**)**.

KC/GRO (CXCL1) levels showed substantial variability across treatment groups, with mean values trending higher in the αSyn group and lower in the PBS + NK group. One-way ANOVA revealed a trend toward significance (p = 0.069), though not statistically significant **(**Fig. 5b**)**. Despite this, group means suggest that αSyn exposure elevates KC/GRO, a chemokine associated with neutrophil recruitment, while NK cell treatment in both PBS + NK and PFF + NK groups modestly reduced KC/GRO levels relative to their respective controls **(**Fig. 5b**)**. These reductions did not reach statistical significance in post hoc tests, but the trend is consistent with other findings showing that NK cells attenuate inflammatory chemokine signals. No significant differences were observed in IL-1β levels across treatment conditions, while IL-2 showed a preferential elevation in the PBS + NK group compared to other conditions **(**Fig. 5b**)**, suggesting NK cells primarily stimulate this pathway in non-pathological settings.

These results collectively show that NK cells modulate specific inflammatory pathways in a context-dependent manner, suppressing pro-inflammatory mediators (e.g., TNFα, IL-12p70) and enhancing selected immune regulators (e.g., IL-4, IL-2) predominantly in healthy environments. The ability of NK cells to dampen αSyn–induced cytokine responses suggests a direct immunomodulatory role in neural cultures.

Immunofluorescence imaging revealed intact neuronal networks in PBS controls, with extensive neurite outgrowth and normal morphology. αSyn treatment induced notable neuronal damage, characterized by neurite fragmentation and abnormal cellular aggregates. NK cell addition to αSyn-challenged cultures partially preserved neuronal integrity and network complexity, suggesting neuroprotective effects **(**Fig. 5c**)**.

### αSyn alters NK cell ligand expression and induces senescence-associated profiles in neural and myeloid cells

NK cells, unlike T and B cells, do not undergo somatic gene rearrangement. Instead, they express a diverse repertoire of activating and inhibitory receptors (NKRs) that collectively determine whether they are activated or restrained [38]. Stress-inducible ligands such as retinoic acid early transcripts α-ε (RAE-1 α-ε), murine UL16-binding protein-like transcript 1 (MULT1), and H60 a-c in mice are recognized by the NKG2D receptor on NK cells, triggering cytotoxic responses against damaged or abnormal cells [39]. In contrast, the inhibitory ligand mQa1b engages the NKG2A/CD94 receptor complex on NK cells, delivering suppressive signals that prevent inappropriate NK activation [40]. Changes in the expression of these ligands can modulate NK cell recognition and the clearance of affected neural and myeloid cells.

**Fig. 6:**
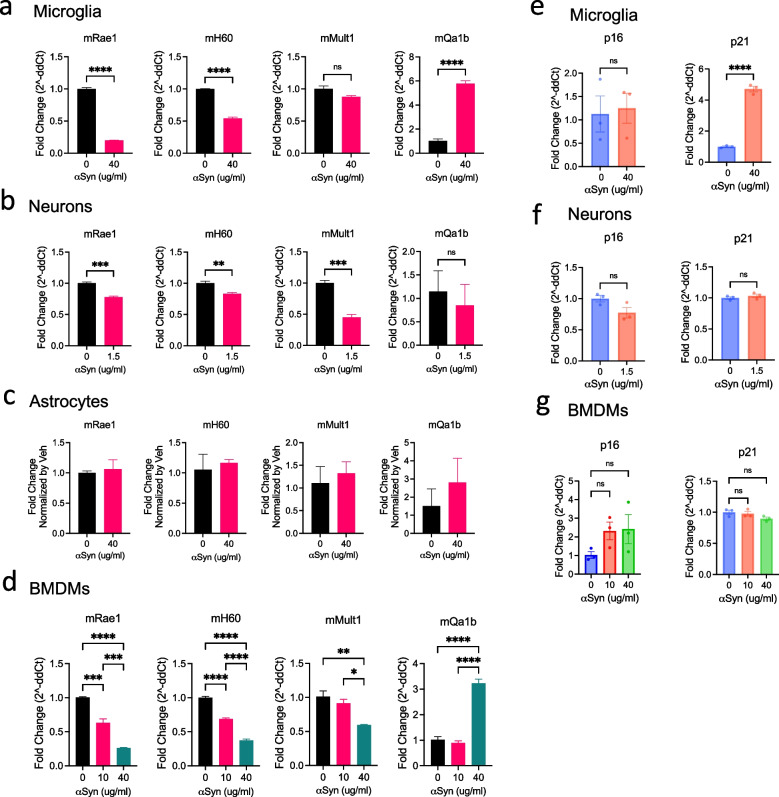
αSyn alters NK cell ligand expression and induces senescence-associated profiles in neural and myeloid cells. (**a-d**) Quantitative analyses of mRNA levels of NK receptor ligands (mRae1, mH60, mMult1, and mQa1b) in primary microglia (a), primary neurons (b), primary astrocytes (c), and BMDM (d) following αSyn treatment. Microglia, astrocytes, and BMDMs were treated with 0, 10, or 40 μg/mL αSyn, while neurons were treated with 0 or 1.5 μg/mL due to their higher sensitivity. Data are presented as fold change (2.^−ΔΔCt^) relative to untreated controls. (**e–g**) Expression of senescence markers p16 and p21 in microglia (e), neurons (f), and BMDMs (g) after αSyn exposure. Data are presented as individual values with mean ± SEM. Statistical analyses were performed using Student’s t-test or one-way ANOVA with Tukey’s post hoc test. *p < 0.05, **p < 0.01, ***p < 0.001, ****p < 0.0001

To investigate whether αSyn pathology impact NK cell surveillance, we assessed the expression of NK cell receptor ligands across major CNS and myeloid cell types. Primary cultures of microglia, neurons, astrocytes, and bone marrow-derived macrophages (BMDMs) were exposed to αSyn, and the expression levels of activating and inhibitory ligands were evaluated. In parallel, we examined the induction of senescence-associated markers, as cellular senescence may promote immune evasion and sustain chronic inflammation during neurodegenerative disease progression **(**Fig. 6**)**. In primary microglia, treatment with αSyn (40 μg/mL) significantly downregulated the activating NK cell ligands mRae1 and mH60 by approximately 85% and 45%, respectively, compared to untreated controls (p < 0.0001). Conversely, the inhibitory ligand mQa1b was upregulated nearly five fold (p < 0.0001), while mMult1 expression remained unchanged **(**Fig. 6a**)**. This ligand shift suggests that αSyn exposure drives microglia toward an NK cell–resistant phenotype.

Additionally, the expression of senescence marker p21 was robustly induced (> fourfold, p < 0.0001), while p16 levels remained unaffected, indicating selective activation of senescence-related pathways **(**Fig. 6e**)**. Neurons, exposed to a lower dose of αSyn (1.5 μg/mL), showed significant downregulation of all activating NK cell ligands (mRae1, mH60, and mMult1; p < 0.01–0.001), suggesting reduced visibility to NK cells **(**Fig. 6b**)**. However, neither the inhibitory ligand mQa1b nor senescence markers (p16, p21) were significantly altered, indicating a cell type–specific and ligand-specific response to αSyn **(**Fig. 6f**)**. Astrocytes demonstrated remarkable resistance to αSyn–induced changes. Even under high-dose αSyn exposure (40 μg/mL), expression of all NK cell receptor ligands remained unchanged **(**Fig. 6c**)**, suggesting that astrocyte–NK cell interactions may remain stable during pathological conditions. BMDMs exhibited the most pronounced and dose-dependent responses to αSyn exposure. Activating ligands mRae1, mH60, and mMult1 were progressively downregulated (p < 0.05–0.0001), while the inhibitory ligand mQa1b was upregulated over threefold at the highest dose (p < 0.0001) **(**Fig. 6d**)**. In contrast to microglia, the senescence markers p16 and p21 exhibited opposite trends: p16 levels increased modestly with αSyn dose, while p21 decreased slightly. However, neither change reached statistical significance, suggesting that αSyn exposure alters NK ligand expression but not senescence-associated markers in BMDMs **(**Fig. 6g**)**.

While the levels of NK cells within the brain parenchyma were low, we included an analysis of NK cell ligand expression in neurons and glial cells to provide broader context. We frame these findings as hypothesis-generating, suggesting that shifts in ligand expression may sensitize glial or neuronal cells to immune modulation, particularly in regions of NK cell access such as meningeal or periventricular sites. Collectively, our data demonstrate that αSyn alters the immunological landscape of neural and myeloid cells by shifting NK receptor ligand expression toward an inhibitory state while selectively inducing senescence markers. These changes are consistent with the possibility that αSyn promotes senescence-like phenotypes that limit immune clearance, thereby contributing to chronic neuroinflammation and persistent pathology in synucleinopathies.

### Systemic NK cell transfer modulates peripheral immunity by increasing follicular B cells and reducing neutrophils without inducing systemic immunotoxicity

**Fig. 7:**
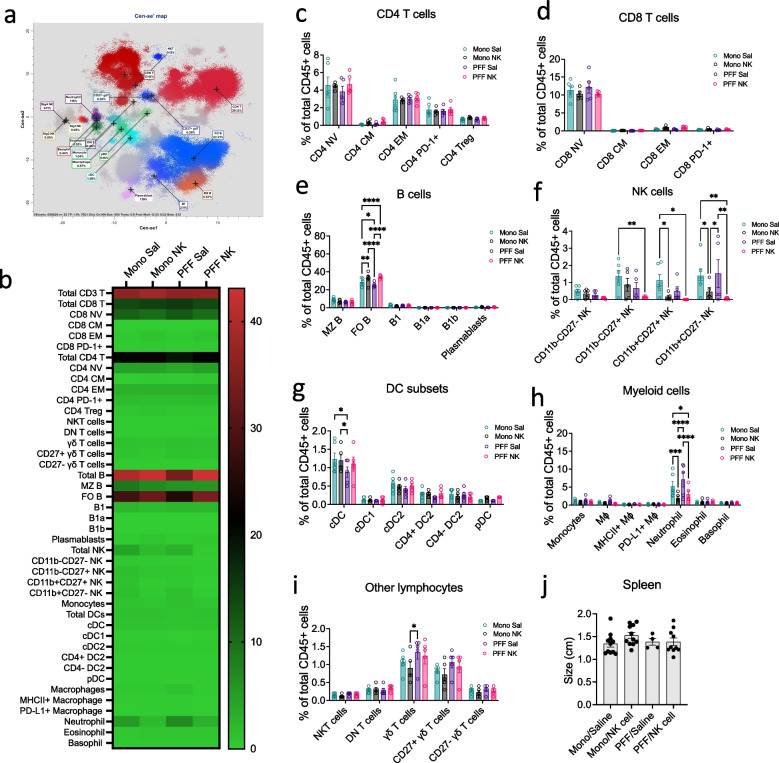
NK cell therapy selectively reduces neutrophil and restores follicular B cells without inducing immunotoxicity at mid-stage disease. M83 Tg mice received unilateral intrastriatal injections of either mono or αSyn PFF and were treated biweekly with saline or NK cells (4 million per dose). At 7 weeks p.i., mice were perfused with PBS; spleens were harvested, processed into single-cell suspensions, pooled, stained with metal-tagged antibodies, and analyzed by CyTOF mass cytometry. (**a**) Cen-se' dimensional reduction map showing clustering of major splenic immune cell populations. **(b)** Heatmap of relative frequencies (%) of immune subsets across experimental groups; color intensity reflects subset percentage (scale 0–40%). (**c–d**) Frequencies of CD4⁺ T cell (c) and CD8⁺ T cell (d) subsets among total CD45⁺ cells, including naïve (NV), central memory (CM), effector memory (EM), PD-1⁺, and regulatory T cells (Tregs, CD4⁺ only). (**e**) B cell subset analysis showing significant expansion of MZ and FO B cells in NK-treated groups. (**f**) Frequencies of NK cell subsets based on CD11b and CD27 expression, confirming NK cell expansion in treated groups. (**g**) DC subset composition, including cDCs (cDC, cDC1, cDC2), CD4⁺ and CD4⁻ DC2s, and plasmacytoid DCs (pDCs). (**h**) Frequencies of myeloid cell populations showing significant reduction in MHCII⁺ macrophages in PFF + NK compared to PFF+Sal groups. **(i)** Other lymphocyte populations, including NKT cells, double-negative (DN) T cells, and γδ T cells. (**j**) Spleen size (cm) showing no significant differences across groups. Data are presented as mean ± SEM (*n* = 5–6 mice per group). Statistical significance was determined by one-way ANOVA with Tukey’s post hoc test. *p < 0.05, **p < 0.01, ***p < 0.001, ****p < 0.0001

To assess the systemic immune effects of NK cell therapy, we performed high-dimensional CyTOF mass cytometry on splenic CD45⁺ cells from the same mice analyzed in Fig. 4**.** The profiling revealed distinct immune cell clusters and treatment-dependent alterations in immune composition **(**Fig. 7a**)**. CD4⁺ T cell subset analysis revealed no significant changes across treatment groups. Frequencies of naïve (CD4⁺ NV), central memory (CD4⁺ CM), effector memory (CD4⁺ EM), PD-1⁺ CD4⁺ T cells, and regulatory T cells (CD4⁺ Tregs) remained consistent regardless of NK cell transfer or αSyn PFF exposure, indicating that neither intervention significantly modulates splenic CD4⁺ T cell composition **(**Fig. 7c**)**. Splenic profiling of CD8⁺ T cell subsets revealed minimal changes across treatment groups. Naïve CD8⁺ T cells (CD8 NV) showed modest variability within groups, with no consistent pattern of expansion or reduction associated with either NK cell transfer or PFF exposure. Central memory (CD8 CM) and effector memory (CD8 EM) subsets were present at low frequencies across all conditions, though a slight increase in CD8 EM cells was noted in NK-treated animals (up to 1.6% in Mono NK and 1.3% in PFF NK), implying a possible trend toward enhanced memory activation with NK therapy **(**Fig. 7d**)**. B cell subset analysis revealed a clear NK cell–mediated modulation, especially within the follicular (FO) B cell compartment. FO B cell frequencies, reduced in PFF mice (22.4–29.9%) compared to Mono (up to 33.6%), were restored and expanded in both Mono + NK and PFF + NK groups (up to 40.5% and 36.7%, respectively), suggesting NK cell-driven support for B cell homeostasis. Plasmablasts also increased in NK-treated groups (up to 1.2%), whereas B1, B1a, and B1b cells remained consistently low across all conditions **(**Fig. 7e**)**. Analysis of splenic NK subsets showed that transferred NK cells skew toward immature phenotypes by day 7 post-infusion. Mature cytotoxic subsets (CD11b⁺CD27⁺ and CD11b⁺CD27⁻) were substantially reduced or nearly absent in NK-treated animals, while immature subsets (CD11b⁻CD27⁻ and CD11b⁻CD27⁺) were more prominent, particularly in Mono NK. PFF + NK animals showed an overall reduction in NK subsets, raising possibility that αSyn pathology may limit NK cell expansion or persistence. These findings align with established literature indicating limited in vivo persistence (~ 7–14 days) of adoptively transferred NK cells **(**Fig. 7f**)**. Classical dendritic cell (cDC) analysis revealed a reduction in total cDC frequencies in PFF-treated mice (0.5–1.2%) compared to mono-injected controls (0.9–1.8%). Notably, cDC levels were restored in PFF + NK-treated mice (0.5–1.7%), suggesting that NK cell therapy supports cDC homeostasis under synucleinopathy conditions. In contrast, the relative distribution of cDC subsets remained consistent across all experimental groups **(**Fig. 7g**)**. Neutrophil frequencies were significantly reduced in NK-treated animals compared to saline-treated controls: Mono + NK (0.8–3.8%) vs. Mono (2.7–10.2%), and PFF + NK (0.9–6.3%) vs. PFF (1.6–11.2%) **(**Fig. 7h**).** This supports the notion that NK cells may suppress neutrophil expansion or infiltration, potentially mitigating pro-inflammatory responses. However, the effect appeared less robust in the PFF + NK group, possibly due to ongoing neuroinflammatory stimuli. Unconventional T cells showed modest treatment-associated changes. NKT cells remained consistently low (~ 0.1–0.2%) across all groups. γδ T cells showed more variability, with a mild expansion in some PFF + NK mice (up to 1.7%), suggesting that NK cells may influence this population in certain inflammatory contexts **(**Fig. 7i**).**

**Fig. 8:**
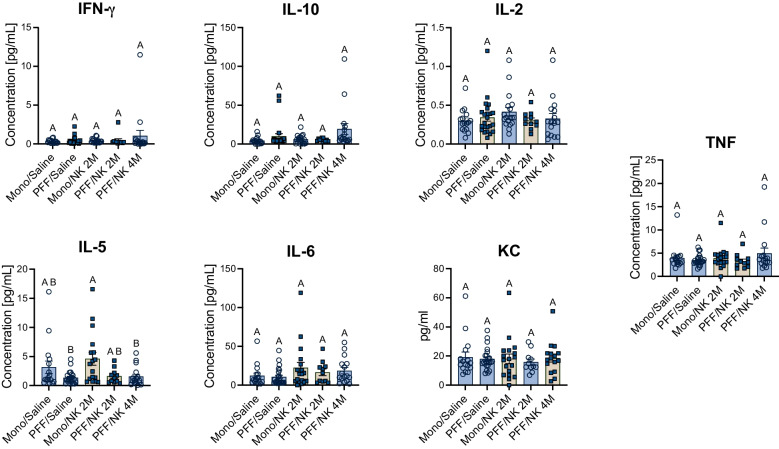
NK cell transfers do not alter systemic cytokine profiles. Serum samples were collected from M83 Tg mice at 11 weeks after intrastriatal injection of mono or αSyn PFF, followed by biweekly saline or NK cells treatments (2 million cells per dose (2M) or 4 million cells per dose (4M)). Cytokine concentrations (pg/mL) were assessed using multiplex assays across the following groups: Mono+Saline, PFF+Saline, Mono + NK 2M, PFF + NK 2M and PFF + NK 4M. Each data point represents an individual mouse. Data were analyzed using one-way ANOVA followed by Tukey’s post hoc test. Different letters indicate statistically significant differences (*p* < 0.05)

Importantly, spleen size did not differ between groups, indicating that NK cell transfer does not induce splenomegaly or systemic immunotoxicity **(**Fig. 7j**)**. Finally, serum cytokine profiling at 11 weeks post-injection revealed no major differences in inflammatory cytokines across groups. While some individual outliers were present, median cytokine levels remained comparable between NK- and saline-treated animals, supporting the immune tolerability of adoptive NK cell therapy **(**Fig. 8**).**

## Discussion

We previously established a progressive synucleinopathy model by inoculating αSyn PFF into M83 Tg mice [27]. These mice developed extensive αSyn pathology and exhibited pronounced motor deficits and nigrostriatal neurodegeneration, making them well-suited for modeling both functional and pathological aspects of disease. Importantly, disease phenotypes emerge within 2–3 months post-injection, enabling timely evaluation of therapeutic interventions such as NK cell therapy. Our results demonstrate that systemic NK cell transfer mitigates key pathological and functional hallmarks of synucleinopathy in the PFF-injected M83 model. NK cell therapy provided dose-dependent motor benefit, as evidenced by reduced hindlimb clasping scores, with the higher dose (4 million cells) conferring the greatest functional improvement. This protective effect was observed during the early to mid-phase of disease progression. However, clinical scoring at the terminal stage (11 weeks) revealed limited efficacy in halting end-stage decline, suggesting that NK cell therapy may be more effective prior to the onset of irreversible neurodegeneration. Pathological assessment confirmed that NK cells modulate αSyn burden in a region- and context-specific manner. In PFF-injected mice, high-dose NK treatment significantly reduced p-αSyn pathology in the SN and brainstem, regions critical for motor control. Conversely, a modest increase in striatal pathology was noted at later time points in PFF + NK mice, possibly reflecting regional differences in NK cell infiltration, immune activation thresholds, or local microenvironmental factors. Interestingly, in the monomer-injected group (a milder model), NK cells reduced pathology across multiple brain regions, suggesting that NK-mediated clearance may be more effective under conditions of lower aggregate burden or earlier disease stages.

In our previous report, this model did not show SNpc dopaminergic neuron loss at the examined time point [27]. A key distinction between that study and the present work lies in the timing of post-injection analysis. In the earlier study, mice were evaluated at 9 weeks after PFF injection, a time point selected because of increased mortality in the NK cell–depleted group. At this stage, dopaminergic neuron loss in the SNpc was not yet reliably detectable. In contrast, adoptive NK cell transfer in the present study enhanced survival and delayed disease progression, allowing us to extend analysis to 11 weeks post-injection. By this later stage, cumulative αSyn pathology and associated neuroinflammation had advanced sufficiently to reveal measurable TH⁺ neuron loss in the SNpc. This distinction underscores the importance of disease stage and observation window in shaping detectable neurodegenerative outcomes in synucleinopathy models, and further suggests that NK cell therapy may mitigate or delay the onset of neuronal loss by altering the tempo of pathology progression.

In the spinal cord, NK cell transfer reduced αSyn pathology most robustly in the lumbar region, with a decreasing gradient toward the cervical region. This rostro-caudal gradient may reflect differential trafficking or functional persistence of transferred NK cells, regional differences in αSyn propagation, or variable accessibility across the CNS axis. Such spatially distinct efficacy could inform region-targeted delivery strategies or adjunctive therapies to enhance global CNS protection.

CD4⁺ T cells are key drivers of neuroinflammation and neurodegeneration in αSyn-based models, promoting myeloid activation and dopaminergic neuron loss, effects mitigated by their depletion [11, 41]. While CD8⁺ T cells also infiltrate the CNS and are abundant at the blood–brain barrier, knockout studies suggest they are not essential for neurodegeneration in some models [11]. Recent studies highlight a protective role for B cells in PD model. Specifically, B cell depletion in the 6-hydroxydopamine (6-OHDA) lesion model led to worsened motor deficits and exacerbated dopaminergic neuron loss [42]. Using high-dimensional CyTOF analysis, we demonstrated that NK cell transfer profoundly influenced both lymphoid and myeloid compartments, revealing a broad immunomodulatory capacity that extends beyond NK cell effector functions alone. Both CD4⁺ and CD8⁺ T cells contribute to PD, though their roles differ across models. One of the most intriguing immunological effects of NK cell therapy was the expansion of CD19⁺ B cells, particularly in the PFF + NK group, suggesting that NK cells may promote B cell recruitment or survival within the inflamed CNS. This was accompanied by a notable shift in T cell composition: an increase in CD8⁺ T cells and a concurrent reduction in CD4⁺ T cells, indicative of a more cytotoxic and potentially immunoregulatory environment. A similar pattern of immune modulation was observed in the periphery. In the spleen, NK cell therapy restored and expanded follicular B cell populations that were otherwise reduced in PFF mice. This supports the idea that NK cells contribute to humoral immune homeostasis, potentially through cytokine signaling or secondary lymphoid organ remodeling.

Neutrophils have been increasingly recognized as key contributors to PD pathogenesis, promoting systemic inflammation, oxidative stress, and blood–brain barrier (BBB) disruption [43]. Elevated neutrophil-to-lymphocyte ratios (NLR), a marker of systemic inflammation, are consistently correlated with PD severity and dopaminergic neurodegeneration [44]. Our findings provide compelling evidence that NK cell therapy directly counteracts this neutrophil-driven inflammatory axis**.** Specifically, we observed a robust reduction in neutrophil frequencies both centrally and peripherally following NK cell transfer, under both mono and PFF αSyn conditions. This implies that NK cells exert broad immunoregulatory effects capable of dampening pro-inflammatory myeloid responses. Importantly, NK cell treatment significantly decreased the abundance of CD11b⁺CD45^high^ cells—activated microglia/macrophages—and restored the homeostatic CD45^low^ microglial pool, suggesting resolution of microglial activation. This is further supported by a marked decrease in MHCII expression, indicating suppressed antigen-presenting activity and possibly attenuated neuroinflammation. Interestingly, CD11c⁺ myeloid populations increased following NK infusion in PFF mice, supporting the emergence of a "disease-associated microglia" (DAM)-like phenotype [45], which has been proposed to play protective roles in neurodegeneration by enhancing phagocytosis and limiting inflammation. The enrichment of this population may reflect a beneficial reprogramming of CNS-resident immune cells. Of note, NK cell transfer in both PFF- and mono-injected mice effectively reestablished baseline homeostatic immune profiles, highlighting its potential to normalize CNS immune dysregulation.

Direct evidence of NK cell engraftment in the brain was confirmed by increased frequencies of CD335⁺ (NKp46⁺) cells following NK cell transfer. Notably, the predominance of immature (CD11b⁻CD27⁻) NK cell phenotypes in both the Mono + NK and PFF + NK groups suggests that NK cells may either undergo phenotypic shifts within the CNS or preferentially infiltrate in a less mature state. Immature NK cells are thought to possess heightened cytokine production with limited cytotoxic activity, features that could be particularly beneficial in the inflamed or degenerating CNS by promoting immunomodulation rather than cytotoxic damage. Thus, the enrichment of CD11b⁻CD27⁻ NK cells in the brain may represent a specialized, neuroprotective subset that adapts to the CNS microenvironment. Alternatively, the reduced NK cell levels observed in PFF + NK mice could reflect impaired CNS homing or diminished persistence under inflammatory conditions, potentially limiting therapeutic durability at later disease stages. Our in vitro data further demonstrated that NK cells exert immunomodulatory effects in the context of αSyn–induced neuroinflammation. Co-culture of NK cells with αSyn PFF-challenged neuron-glia cultures significantly altered cytokine profiles, supporting the idea that NK cells actively reshape the inflammatory environment. This may reflect efforts to resolve inflammation without promoting compensatory immune suppression.

NK cells play a regulatory role in CNS inflammation by targeting autoimmunogenic T cells and activated microglia with reduced expression of Qa-1, the murine homolog of HLA-E, which normally binds the inhibitory NKG2A receptor on NK cells to suppress cytotoxicity [46, 47]. NK cells also mediate cytotoxicity against immature or damaged neurons, including dorsal root ganglion neurons that express RAE1, a ligand for the activating NKG2D receptor [48, 49]. We demonstrated that αSyn exposure induced a marked shift in NK ligand expression across multiple cell types: activating ligands such as Rae1, H60, and Mult1 were significantly downregulated, while the inhibitory ligand mQa1b was upregulated—particularly in microglia and BMDMs. This pattern suggests that αSyn pathology promotes an immunoevasive phenotype that may impair NK cell–mediated clearance of damaged or senescent cells. These findings align with the broader concept of immune privilege in chronic neurodegeneration, where persistent inflammation paradoxically dampens immune surveillance and contributes to disease progression.

In primary microglia exposed to αSyn, p21-but not p16-was upregulated, consistent with prior reports showing αSyn PFF-induced p21 elevation in reactive glia in mouse models [50]. This selective induction points to a partial senescence-like state, which may sustain chronic inflammation and impair immune clearance without triggering full cellular senescence. Such a phenotype aligns with the concept of a senescence-associated secretory profile (SASP) and may have important implications for persistent neuroinflammation in synucleinopathies. Our analysis of NK cell ligands should be considered hypothesis-generating rather than definitive evidence of direct NK–glia or NK–neuron interactions within the parenchyma. Instead, the observed shifts in ligand expression may reflect changes that sensitize glial or neuronal cells to immune modulation at CNS border interfaces (e.g., meninges, perivascular regions, or ventricular sites), where NK cells are more likely to exert regulatory influence. Based on these findings, we propose that the progressive accumulation of senescence-like cells, coupled with persistent neuroinflammation, may underlie the reduced effectiveness of NK cell therapies at later disease stages. Notably, despite substantial immunological reprogramming in the CNS and periphery, serum levels of inflammatory cytokines remained largely unchanged across treatment groups. The absence of systemic cytokine storm or broad inflammatory signatures supports the tolerability and translational potential of repeated NK cell dosing.

## Conclusion

Collectively, our findings highlight NK cells as multifaceted immunomodulators capable of targeting disease-relevant mechanisms across both central and peripheral compartments. By attenuating αSyn pathology, suppressing maladaptive inflammation, and restoring immune balance without inducing systemic toxicity, NK cell therapy emerges as a promising, well-tolerated, and mechanistically targeted intervention for synucleinopathies. Further studies are warranted to enhance NK cell persistence, optimize dosing strategies, and explore combinatorial approaches to achieve durable disease modification.

## Supplementary Information

Below is the link to the electronic supplementary material.

Supplementary file1 (PDF 1605 KB)

## Data Availability

The data supporting the findings of this study are available from the corresponding authors upon request.

## Abbreviations

BBB: Blood-brain barrier
BMDMs: Bone marrow-derived macrophages
CD: Cluster of differentiation
CM: Central memory
CNS: Central nervous system
CSF: Cerebrospinal fluid
CyTOF: Cytometry by time of flight (Mass cytometry)
DAMs: Disease-associated microglia
EM: Effector memory
FO: Follicular
HLA-E: Human leukocyte antigen-E
H60: Histocompatibility 60
IL: Interleukin
KC/GRO: Keratinocyte chemoattractant/Growth-regulated oncogene
MHC II: Major Histocompatibility Complex II
mQa1b: Murine homolog of HLA-E
MULT1: Murine UL 16-binding protein-like transcript 1
NK: Natural killer
NKG2A or NKG2D: Natural killer group 2 member A or D protein
NLR: Neutrophil-to-lymphocyte Ratios
NKT: Natural Killer T cells
NV: Naïve
PD: Parkinson’s disease
PFF αSyn: Preformed fibril α-synuclein
PK: Proteinase K
p-αSyn: Phosphorylated α-synuclein at serine 129
RAE-1: Retinoic acid early inducible 1
SASP: Senescence-associated secretory profile
SN: Substantia nigra
SNpc: Substantia nigra pars compacta
STR: Striatum
TCR: T-cell receptor
TEM: Transmission electron microscopy
Tg: Transgenic
TH: Tyrosine Hydroxylase
TLR: Toll-like receptor
TNF: Tumor necrosis factor
Tregs: Regulatory T cells

## References

[1] Kalia LV, Lang AE. Parkinson’s disease. Lancet. 2015;386(9996):896–912. 25904081 10.1016/S0140-6736(14)61393-3

[2] Spillantini MG, Crowther RA, Jakes R, Hasegawa M, Goedert M. α-Synuclein in filamentous inclusions of Lewy bodies from Parkinson’s disease and dementia with Lewy bodies. Proc Natl Acad Sci. 1998;95(11):6469–73. 9600990 10.1073/pnas.95.11.6469 PMC27806

[3] Sulzer D, Surmeier DJ. Neuronal vulnerability, pathogenesis, and Parkinson’s disease. Mov Disord. 2013;28(6):715–24. 23589357 10.1002/mds.25187

[4] Bloem BR, Okun MS, Klein C. Parkinson’s disease. Lancet. 2021;397(10291):2284–303. 33848468 10.1016/S0140-6736(21)00218-X

[5] Armstrong MJ, Okun MS. Diagnosis and treatment of Parkinson disease: a review. JAMA. 2020;323(6):548–60. 32044947 10.1001/jama.2019.22360

[6] Schapira AH, Chaudhuri KR, Jenner P. Non-motor features of Parkinson disease. Nat Rev Neurosci. 2017;18(7):435–50. 28592904 10.1038/nrn.2017.62

[7] Reale M, Iarlori C, Thomas A, Gambi D, Perfetti B, Di Nicola M, et al. Peripheral cytokines profile in Parkinson’s disease. Brain Behav Immun. 2009;23(1):55–63. 18678243 10.1016/j.bbi.2008.07.003

[8] Williams-Gray C. Wijeyekoon R1, Yarnall AJ2, Lawson RA2, Breen DP1, Evans JR1, Cummins GA1, Duncan GW3, Khoo TK4, Burn DJ2, Barker RA1; ICICLE-PD study group. Serum immune markers and disease progression in an incident Parkinson's disease cohort (ICICLE-PD). Mov Disord. 2016;31(7):995–1003. 10.1002/mds.26563 PMC4957620 26999434

[9] Béraud D, Hathaway HA, Trecki J, Chasovskikh S, Johnson DA, Johnson JA, et al. Microglial activation and antioxidant responses induced by the Parkinson’s disease protein α-synuclein. J Neuroimmune Pharmacol. 2013;8:94–117. 23054368 10.1007/s11481-012-9401-0 PMC3582877

[10] Baba Y, Kuroiwa A, Uitti RJ, Wszolek ZK, Yamada T. Alterations of t-lymphocyte populations in Parkinson disease. Parkinsonism Relat Disord. 2005;11(8):493–8. 16154792 10.1016/j.parkreldis.2005.07.005

[11] Williams GP, Schonhoff AM, Jurkuvenaite A, Gallups NJ, Standaert DG, Harms AS. CD4 t cells mediate brain inflammation and neurodegeneration in a mouse model of Parkinson’s disease. Brain. 2021;144(7):2047–59. 33704423 10.1093/brain/awab103 PMC8370411

[12] Scott KM, Chong YT, Park S, Wijeyekoon RS, Hayat S, Mathews RJ, et al. B lymphocyte responses in Parkinson’s disease and their possible significance in disease progression. Brain Commun. 2023;5(2):fcad060. 36993946 10.1093/braincomms/fcad060 PMC10042276

[13] Poli A, Kmiecik J, Domingues O, Hentges F, Blery M, Chekenya M, et al. NK cells in central nervous system disorders. J Immunol. 2013;190(11):5355–62. 23687193 10.4049/jimmunol.1203401

[14] Small CL, McCormick S, Gill N, Kugathasan K, Santosuosso M, Donaldson N, et al. NK cells play a critical protective role in host defense against acute extracellular *Staphylococcus aureus* bacterial infection in the lung. J Immunol. 2008;180(8):5558–68. 18390740 10.4049/jimmunol.180.8.5558

[15] Schmidt RL, Filak HC, Lemon JD, Potter TA, Lenz LL. A lysm and SH3-domain containing region of the *Listeria monocytogenes* p60 protein stimulates accessory cells to promote activation of host NK cells. PLoS Pathog. 2011;7(11):e1002368. 22072975 10.1371/journal.ppat.1002368 PMC3207947

[16] Sagiv A, Biran A, Yon M, Simon J, Lowe SW, Krizhanovsky V. Granule exocytosis mediates immune surveillance of senescent cells. Oncogene. 2013;32(15):1971–7. 22751116 10.1038/onc.2012.206 PMC3630483

[17] Thoren FB, Riise RE, Ousback J, Della Chiesa M, Alsterholm M, Marcenaro E, et al. Human NK cells induce neutrophil apoptosis via an NKp46- and Fas-dependent mechanism. J Immunol. 2012;188(4):1668–74. 22231698 10.4049/jimmunol.1102002

[18] Waggoner SN, Kumar V. Evolving role of 2B4/CD244 in T and NK cell responses during virus infection. Front Immunol. 2012;3:377. 23248626 10.3389/fimmu.2012.00377 PMC3518765

[19] Martin-Fontecha A, Thomsen LL, Brett S, Gerard C, Lipp M, Lanzavecchia A, et al. Induced recruitment of NK cells to lymph nodes provides IFN-gamma for T(H)1 priming. Nat Immunol. 2004;5(12):1260–5. 15531883 10.1038/ni1138

[20] Vitale M, Della Chiesa M, Carlomagno S, Pende D, Arico M, Moretta L, et al. NK-dependent DC maturation is mediated by TNFalpha and IFNgamma released upon engagement of the NKp30 triggering receptor. Blood. 2005;106(2):566–71. 15784725 10.1182/blood-2004-10-4035

[21] Li Q, Cheng Z, Zhou L, Darmanis S, Neff NF, Okamoto J, et al. Developmental heterogeneity of microglia and brain myeloid cells revealed by deep single-cell RNA sequencing. Neuron. 2019;101(2):207-23 e10. 30606613 10.1016/j.neuron.2018.12.006 PMC6336504

[22] Van Hove H, Martens L, Scheyltjens I, De Vlaminck K, Pombo Antunes AR, De Prijck S, et al. A single-cell atlas of mouse brain macrophages reveals unique transcriptional identities shaped by ontogeny and tissue environment. Nat Neurosci. 2019. 10.1038/s41593-019-0393-4. 31061494 10.1038/s41593-019-0393-4

[23] Mihara T, Nakashima M, Kuroiwa A, Akitake Y, Ono K, Hosokawa M, et al. Natural killer cells of Parkinson’s disease patients are set up for activation: a possible role for innate immunity in the pathogenesis of this disease. Parkinsonism Relat Disord. 2008;14(1):46–51. 17702627 10.1016/j.parkreldis.2007.05.013

[24] Niwa F, Kuriyama N, Nakagawa M, Imanishi J. Effects of peripheral lymphocyte subpopulations and the clinical correlation with Parkinson’s disease. Geriatr Gerontol Int. 2012;12(1):102–7. 21929737 10.1111/j.1447-0594.2011.00740.x

[25] Chen L, Zuo Y, Zhu L, Zhang Y, Li S, Ma F, et al. Peripheral venous blood neutrophil-to-lymphocyte ratio predicts survival in patients with advanced gastric cancer treated with neoadjuvant chemotherapy. Onco Targets Ther. 2017;10:2569–80. 28553122 10.2147/OTT.S134716 PMC5440079

[26] Earls RH, Menees KB, Chung J, Barber J, Gutekunst CA, Hazim MG, et al. Intrastriatal injection of preformed alpha-synuclein fibrils alters central and peripheral immune cell profiles in non-transgenic mice. J Neuroinflammation. 2019;16(1):250. 31796095 10.1186/s12974-019-1636-8 PMC6889316

[27] Earls RH, Menees KB, Chung J, Gutekunst CA, Lee HJ, Hazim MG, et al. NK cells clear alpha-synuclein and the depletion of NK cells exacerbates synuclein pathology in a mouse model of alpha-synucleinopathy. Proc Natl Acad Sci U S A. 2020;117(3):1762–71. 31900358 10.1073/pnas.1909110117 PMC6983411

[28] Earls RH, Menees KB, Chung J, Gutekunst C-A, Lee HJ, Hazim MG, et al. NK cells clear α-synuclein and the depletion of NK cells exacerbates synuclein pathology in a mouse model of α-synucleinopathy. Proc Natl Acad Sci. 2020;117(3):1762–71. 31900358 10.1073/pnas.1909110117 PMC6983411

[29] Giasson BI, Murray IV, Trojanowski JQ, Lee VM. A hydrophobic stretch of 12 amino acid residues in the middle of alpha-synuclein is essential for filament assembly. J Biol Chem. 2001;276(4):2380–6. 11060312 10.1074/jbc.M008919200

[30] Rutherford NJ, Sacino AN, Brooks M, Ceballos-Diaz C, Ladd TB, Howard JK, et al. Studies of lipopolysaccharide effects on the induction of alpha-synuclein pathology by exogenous fibrils in transgenic mice. Mol Neurodegener. 2015;10:32. 26223783 10.1186/s13024-015-0029-4 PMC4520273

[31] Lei P, Ayton S, Moon S, Zhang Q, Volitakis I, Finkelstein DI, et al. Motor and cognitive deficits in aged tau knockout mice in two background strains. Mol Neurodegener. 2014;9:1–12. 25124182 10.1186/1750-1326-9-29 PMC4141346

[32] Lee JK, Tansey MG. Microglia isolation from adult mouse brain. Methods Mol Biol. 2013;1041:17–23. 23813365 10.1007/978-1-62703-520-0_3 PMC4145600

[33] Lee JK, Chung J, Kannarkat GT, Tansey MG. Critical role of regulator G-protein signaling 10 (RGS10) in modulating macrophage M1/M2 activation. PLoS ONE. 2013;8(11):e81785. 24278459 10.1371/journal.pone.0081785 PMC3836764

[34] Zhang Y, Wallace DL, de Lara CM, Ghattas H, Asquith B, Worth A, et al. In vivo kinetics of human natural killer cells: the effects of ageing and acute and chronic viral infection. Immunology. 2007;121(2):258–65. 17346281 10.1111/j.1365-2567.2007.02573.x PMC2265941

[35] Prlic M, Blazar BR, Farrar MA, Jameson SC. In vivo survival and homeostatic proliferation of natural killer cells. J Exp Med. 2003;197(8):967–76. 12695488 10.1084/jem.20021847 PMC2193876

[36] Wang JW, Howson JM, Ghansah T, Desponts C, Ninos JM, May SL, et al. Influence of SHIP on the NK repertoire and allogeneic bone marrow transplantation. Science. 2002;295(5562):2094–7. 11896280 10.1126/science.1068438

[37] Ohira M, Ohdan H, Mitsuta H, Ishiyama K, Tanaka Y, Igarashi Y, et al. Adoptive transfer of TRAIL-expressing natural killer cells prevents recurrence of hepatocellular carcinoma after partial hepatectomy. Transplantation. 2006;82(12):1712–9. 17198265 10.1097/01.tp.0000250935.41034.2d

[38] Earls RH, Lee JK. The role of natural killer cells in Parkinson’s disease. Exp Mol Med. 2020;52(9):1517–25. 32973221 10.1038/s12276-020-00505-7 PMC8080760

[39] Carapito R, Bahram S. Genetics, genomics, and evolutionary biology of NKG2D ligands. Immunol Rev. 2015;267(1):88–116. 26284473 10.1111/imr.12328

[40] Vance RE, Kraft JR, Altman JD, Jensen PE, Raulet DH. Mouse CD94/NKG2A is a natural killer cell receptor for the nonclassical major histocompatibility complex (MHC) class I molecule Qa-1(b). J Exp Med. 1998;188(10):1841–8. 9815261 10.1084/jem.188.10.1841 PMC2212405

[41] Mamula D, Khosousi S, He Y, Lazarevic V, Svenningsson P. Impaired migratory phenotype of CD4(+) T cells in Parkinson’s disease. NPJ Parkinsons Dis. 2022;8(1):171. 36496415 10.1038/s41531-022-00438-0 PMC9741605

[42] Scott KM, Chong YT, Park S, Wijeyekoon RS, Hayat S, Mathews RJ, et al. B lymphocyte responses in Parkinson’s disease and their possible significance in disease progression. Brain Commun. 2023;5(2):fcad060. 36993946 10.1093/braincomms/fcad060 PMC10042276

[43] Santos-Lima B, Pietronigro EC, Terrabuio E, Zenaro E, Constantin G. The role of neutrophils in the dysfunction of central nervous system barriers. Front Aging Neurosci. 2022;14:965169. 36034148 10.3389/fnagi.2022.965169 PMC9404376

[44] Hosseini S, Shafiabadi N, Khanzadeh M, Ghaedi A, Ghorbanzadeh R, Azarhomayoun A, et al. Neutrophil to lymphocyte ratio in Parkinson’s disease: a systematic review and meta-analysis. BMC Neurol. 2023;23(1):333. 37735638 10.1186/s12883-023-03380-7 PMC10512499

[45] Keren-Shaul H, Spinrad A, Weiner A, Matcovitch-Natan O, Dvir-Szternfeld R, Ulland TK, et al. A unique microglia type associated with restricting development of alzheimer’s disease. Cell. 2017;169(7):1276-90 e17. 28602351 10.1016/j.cell.2017.05.018

[46] Lu L, Ikizawa K, Hu D, Werneck MB, Wucherpfennig KW, Cantor H. Regulation of activated CD4+ T cells by NK cells via the Qa-1-NKG2A inhibitory pathway. Immunity. 2007;26(5):593–604. 17509909 10.1016/j.immuni.2007.03.017 PMC3428267

[47] Leavenworth JW, Schellack C, Kim HJ, Lu L, Spee P, Cantor H. Analysis of the cellular mechanism underlying inhibition of EAE after treatment with anti-NKG2A F(ab’)2. Proc Natl Acad Sci U S A. 2010;107(6):2562–7. 20133787 10.1073/pnas.0914732107 PMC2823885

[48] Backstrom E, Chambers BJ, Kristensson K, Ljunggren HG. Direct NK cell-mediated lysis of syngenic dorsal root ganglia neurons in vitro. J Immunol. 2000;165(9):4895–900. 11046014 10.4049/jimmunol.165.9.4895

[49] Davies AJ, Kim HW, Gonzalez-Cano R, Choi J, Back SK, Roh SE, et al. Natural killer cells degenerate intact sensory afferents following nerve injury. Cell. 2019;176(4):716-28 e18. 30712871 10.1016/j.cell.2018.12.022 PMC6418410

[50] Verma DK, Seo BA, Ghosh A, Ma SX, Hernandez-Quijada K, Andersen JK, et al. Alpha-synuclein preformed fibrils induce cellular senescence in Parkinson’s disease models. Cells. 2021. 10.3390/cells10071694. 34359864 10.3390/cells10071694 PMC8304385

