# Supplementary material for: Adoptive NK cell transfer confers neuroprotection by attenuating neuroinflammation and alpha-synuclein pathology in a mouse model of synucleinopathy
Source: Mol Neurodegener Adv. 2025 Nov 27;1(1):6. doi: 10.1186/s44477-025-00006-9 (PMC12672819; doi:10.1186/s44477-025-00006-9)
Supplement: Supplementary file 1 — Supplementary file1 (PDF 1605 KB) [file 44477_2025_6_MOESM1_ESM.pdf]

Supplement Figure S1

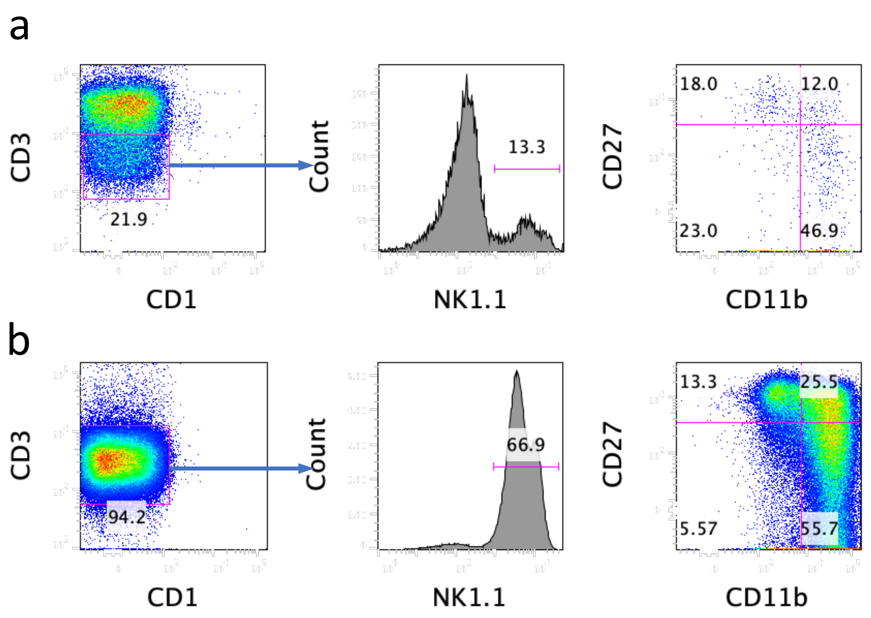

Supplementary Figure 1. Phenotypic characterization of untouched NK cells isolated from B6C3H donor mice. (a) Flow cytometry analysis prior to NK enrichment. Cells were first gated on singlets and $CD45^+$ leukocytes, followed by identification of NK cells as CD3-$NK1.1^+$. NK cell subsets were determined based on CD11b and CD27 expression. (b) Flow cytometry following negative selection (untouched NK cell enrichment) and immediately prior to adoptive transfer. NK cell subsets were again classified as immature ($CD11b^-CD27^+$), early mature ($CD11b^+CD27^+$), terminally mature ($CD11b^+CD27^-$), and double-negative ($CD11b^-CD27^-$) populations. The resulting NK cell product demonstrated a distribution of maturation states consistent with effective enrichment and immunological heterogeneity relevant to downstream functional outcomes.

Supplement Figure S2

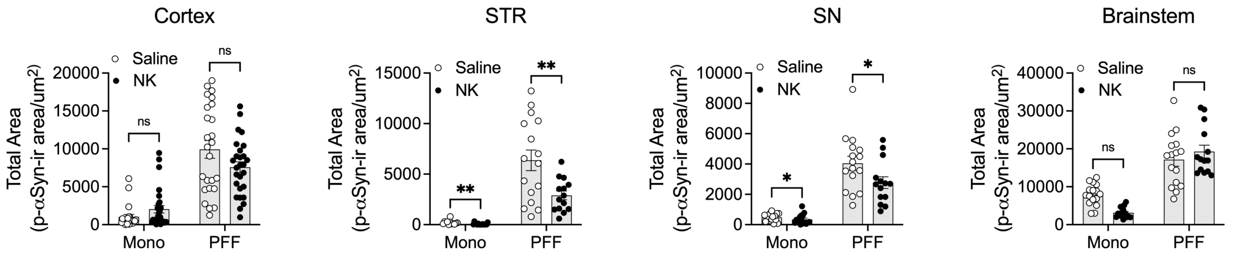

Supplementary Figure 2. NK cell infusion at lower dose mitigates pathological α-synuclein burden in specific brain regions. (a) M83 Tg mice were treated a lower dose of NK cells (2 million NK cells, i.p.) or saline every two weeks following intrastriatal injection of either the monomer or PFF αSyn. At 12 weeks post-injection (p.i.) brains were collected and immunohistochemictry for PK-resistant pS129-aSyn were performed. Graphs show the average total area of pS129-aSyn-positive inclusions per region of interest in the cortex, striatum, SN, and brainstem. Quantification was performed on immunohistological sections from mice in each treated group (n = 4–6 mice per group). Statistical analysis was conducted using two-way ANOVA followed by Fisher's LSD post hoc test. *p < 0.05, **p < 0.01 comparing saline- vs. NK-treated groups. Error bars represent mean ± SEM.

Supplement Figure S3

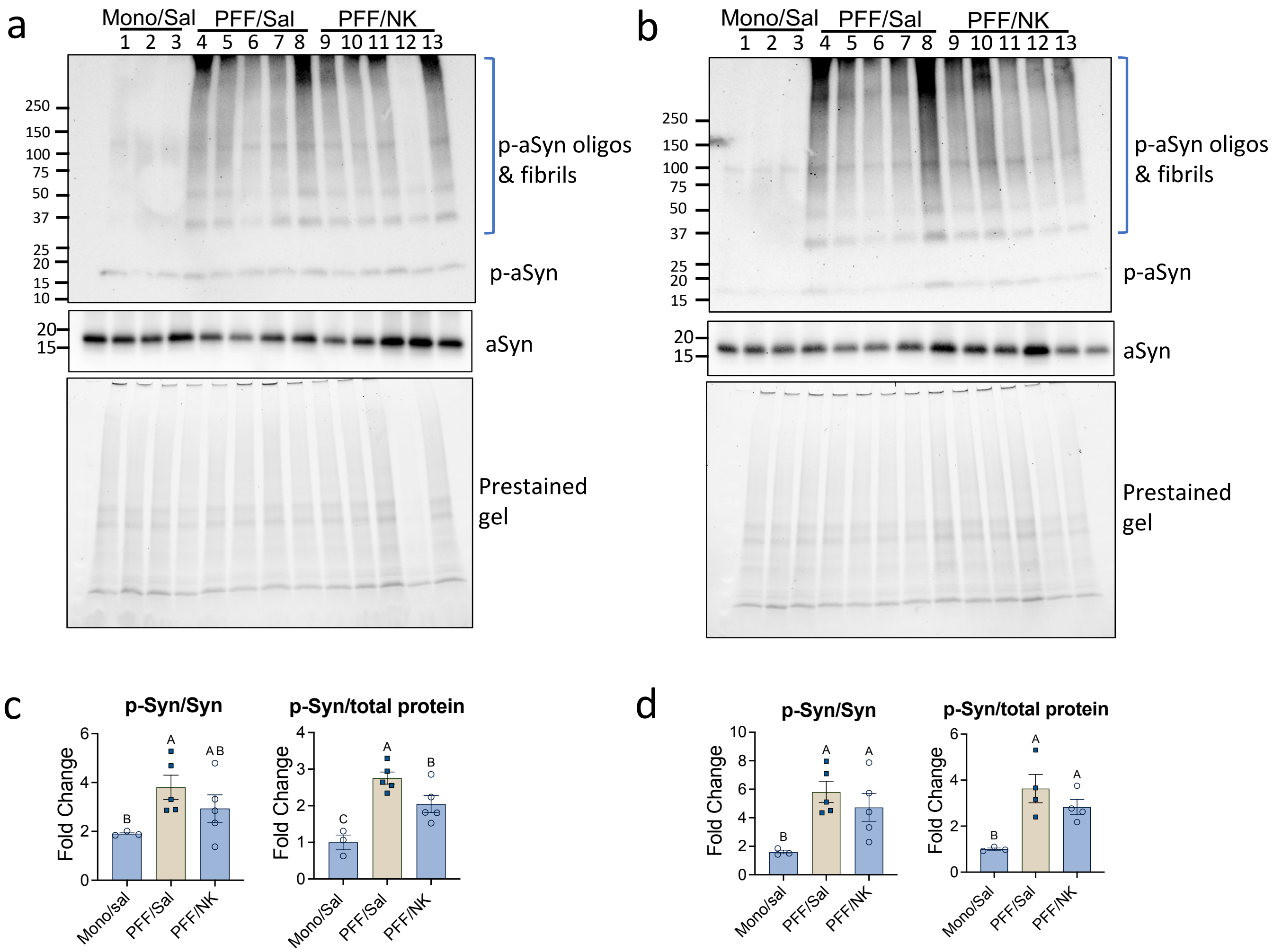

Supplementary Figure 3. NK cell infusion mitigates pathological α-synuclein accumulation in the spinal cord of M83 transgenic mice following PFF injection. M83 mice (8 weeks old) received unilateral stereotaxic injections of either monomeric (Mono) or aSyn PFF into the striatum. Mice were treated with either saline or 4 million NK cells via i.p. injection every two weeks and sacrificed at 7 weeks post-injection. Representative immunoblot images of p-aSyn in the thoracic (a) and cervical (b) spinal cords. Samples were collected from M83 Tg mice in the PFF α-syn and Mono α-syn saline groups (n = 3–5 mice per group).
